# Clonal hematopoiesis is associated with protection from Alzheimer’s disease

**DOI:** 10.1038/s41591-023-02397-2

**Published:** 2023-06-15

**Authors:** Hind Bouzid, Julia A. Belk, Max Jan, Yanyan Qi, Chloé Sarnowski, Sara Wirth, Lisa Ma, Matthew R. Chrostek, Herra Ahmad, Daniel Nachun, Winnie Yao, NHLBI Trans-Omics for Precision Medicine (TOPMed) Consortium, Joshua Bis, Bruce Psaty, Alexa Beiser, Alexander G. Bick, Joshua C. Bis, Myriam Fornage, William T. Longstreth, Oscar L. Lopez, Pradeep Natarajan, Bruce M. Psaty, Claudia L. Satizabal, Joshua Weinstock, Eric B. Larson, Paul K. Crane, C. Dirk Keene, Sudha Seshadri, Ansuman T. Satpathy, Thomas J. Montine, Siddhartha Jaiswal

**Affiliations:** 1 Department of Pathology, Stanford University School of Medicine, Stanford, CA USA; 2 Department of Computer Science, Stanford University, Stanford, CA USA; 3 Department of Pathology, Massachusetts General Hospital, Boston, MA USA; 4 Department of Epidemiology, Human Genetics and Environmental Sciences, The University of Texas Health Science Center at Houston, School of Public Health, Houston, TX USA; 5 Department of Cardiology, Charité Universitätsmedizin, Berlin, Germany; 6 Department of Biostatistics, Boston University School of Public Health, Boston, MA USA; 7 Department of Medicine, Vanderbilt University Medical Center, Nashville, TN USA; 8 Cardiovascular Health Research Unit, Department of Medicine, University of Washington, Seattle, WA USA; 9 Brown Foundation Institute of Molecular Medicine, McGovern Medical School, University of Texas Health Science Center at Houston, Houston, TX USA; 10 Departments of Neurology and Epidemiology, University of Washington, Seattle, WA USA; 11 Department of Neurology, University of Pittsburgh School of Medicine, Pittsburgh, PA USA; 12 Medical and Population Genetics and Cardiovascular Disease Initiative, Broad Institute of Harvard and MIT, Cambridge, MA USA; 13 Cardiovascular Research Center, Massachusetts General Hospital, Harvard Medical School, Boston, MA USA; 14 Cardiovascular Health Research Unit, Departments of Medicine, Epidemiology, and Health Systems and Population Health, University of Washington, Seattle, WA USA; 15 Glenn Biggs Institute for Alzheimer’s & Neurodegenerative Diseases, University of Texas Health Science Center, San Antonio, TX USA; 16 Department of Population Health Sciences, University of Texas Health Science Center, San Antonio, TX USA; 17 Department of Neurology, Boston University School of Medicine, Boston, MA USA; 18 Center for Statistical Genetics, Department of Biostatistics, University of Michigan School of Public Health, Ann Arbor, MI USA; 19 Kaiser Permanente Washington Health Research Institute, Seattle, WA USA; 20 Department of Medicine, University of Washington, Seattle, WA USA; 21 Department of Laboratory Medicine and Pathology, University of Washington, Seattle, WA USA; 22 Institute for Stem Cell Biology and Regenerative Medicine, Stanford University School of Medicine, Stanford, CA USA; 23 The Phil & Penny Knight Initiative for Brain Resilience at the Wu Tsai Neurosciences Institute, Stanford University, Stanford, CA USA

**Keywords:** Immunology, Genetic association study

## Abstract

Clonal hematopoiesis of indeterminate potential (CHIP) is a premalignant expansion of mutated hematopoietic stem cells. As CHIP-associated mutations are known to alter the development and function of myeloid cells, we hypothesized that CHIP may also be associated with the risk of Alzheimer’s disease (AD), a disease in which brain-resident myeloid cells are thought to have a major role. To perform association tests between CHIP and AD dementia, we analyzed blood DNA sequencing data from 1,362 individuals with AD and 4,368 individuals without AD. Individuals with CHIP had a lower risk of AD dementia (meta-analysis odds ratio (OR) = 0.64, *P* = 3.8 × 10^−5^), and Mendelian randomization analyses supported a potential causal association. We observed that the same mutations found in blood were also detected in microglia-enriched fraction of the brain in seven of eight CHIP carriers. Single-nucleus chromatin accessibility profiling of brain-derived nuclei in six CHIP carriers revealed that the mutated cells comprised a large proportion of the microglial pool in the samples examined. While additional studies are required to validate the mechanistic findings, these results suggest that CHIP may have a role in attenuating the risk of AD.

## Abstract (web summary)

Clonal hematopoiesis of indeterminate potential (CHIP) was found to be associated with a protective effect from Alzheimer’s disease (AD) in large population-based cohorts.

## Main

Clonal hematopoiesis of indeterminate potential (CHIP) is an age-associated expansion of hematopoietic stem cells (HSCs) found in 10–30% of those older than 70 (refs. ^1–4^). It most commonly occurs due to truncating or loss-of-function mutations in transcriptional regulators such as *DNMT3A*, *TET2* and *ASXL1* and can be detected by sequencing of DNA from peripheral blood or bone marrow cells^5^. As these are also founding mutations for hematological neoplasms such as acute myeloid leukemia, it is unsurprising that CHIP associates with a higher risk of developing these cancers^1,2,6,7^. However, CHIP also associates with an increased risk of atherosclerotic cardiovascular disease and death^8–10^. This link is believed to be causal as mice that are deficient for *Tet2* or *Dnmt3a* in hematopoietic cells develop more severe cardiovascular phenotypes, possibly due to altered gene expression in mutant macrophages, which favors the more rapid progression of the lesions^8,11,12^.

Alzheimer’s disease (AD) remains a leading cause of morbidity and mortality in the elderly, but therapies that can effectively slow or halt its progression are lacking. Genome-wide association studies (GWAS) have implicated functional alterations of microglia (MG), the macrophage-like hematopoietic cells in the brain, as a major driver of AD risk^13^. Because CHIP-associated mutations influence the function of myeloid cells^8,11,14^, we tested whether CHIP was associated with the risk of AD.

## Results

### Association between CHIP and AD dementia

To test the association between CHIP and incident AD dementia, we used data from the Framingham Heart Study (FHS) and the Cardiovascular Health Study (CHS), which are two cohorts within the Trans-omics for Precision Medicine (TOPMed) project^15^. CHIP variants (Supplementary Table 1) were identified from blood-derived whole-genome sequencing (WGS) data as previously described^9^. AD dementia was diagnosed when participants met the criteria of the National Institute of Neurological and Communicative Disorders and Stroke (NINCDS) and the AD and Related Disorders Association (ADRDA) for definite, probable or possible AD.

**Fig. 1:**
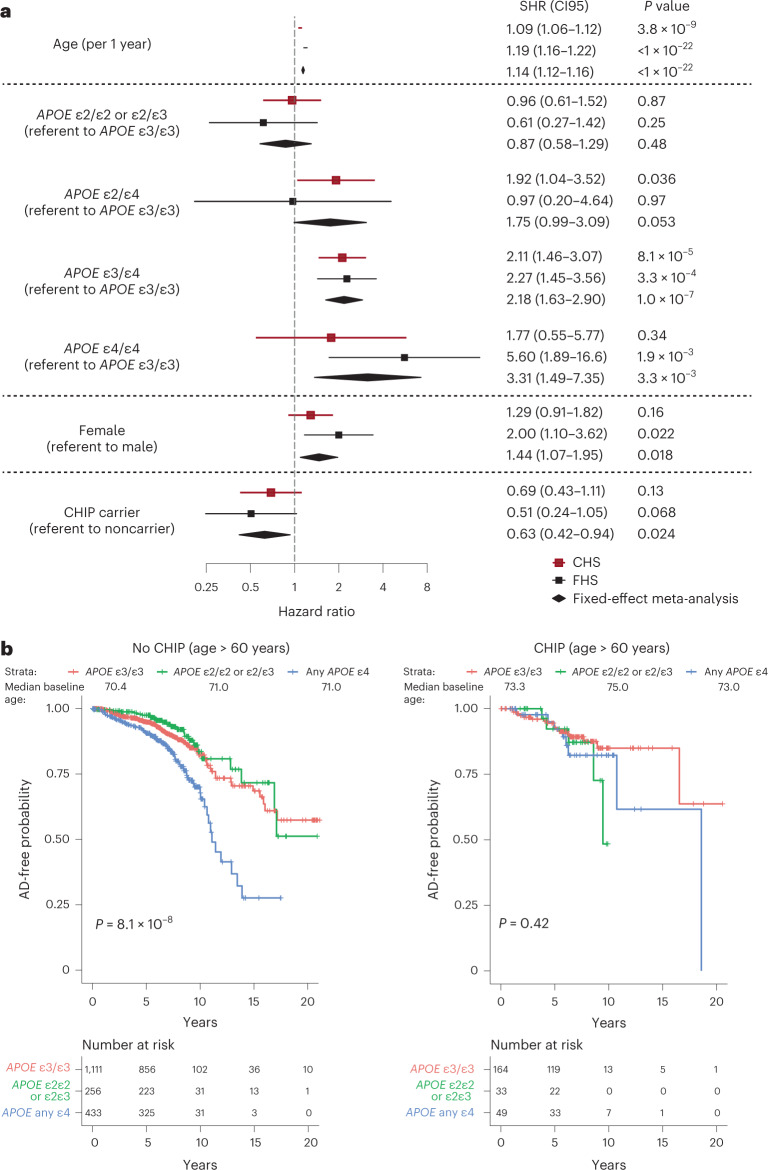
Association of CHIP and AD in longitudinal cohorts. **a**, Effect of CHIP on the risk of incident AD in CHS and FHS. SHR, CI95 and two-sided Wald *P* values were calculated for each covariate from CRR models, which included age at blood draw, sex and *APOE* genotype as covariates. The measure of center is the SHR and the lengths of the lines represent the CI95 for the SHR. Results from CHS and FHS were then meta-analyzed using a fixed-effects model for the two cohorts, the center of the diamonds represents the SHR and the lengths represent the CI95 for the SHR. People in FHS, *n* = 2,437 and people in CHS, *n* = 743. **b**, Kaplan–Meier curve showing AD-free probability in CHIP noncarriers (left) and carriers (right), stratified by *APOE* genotype. Analysis was restricted to those in FHS and CHS older than 60 years at the time of blood draw, and the results of a two-sided log-rank test are shown. People for non-CHIP carriers, *n* = 1,800 and people for CHIP carriers, *n* = 246. CI95, 95% confidence interval.

After excluding those with coronary heart disease, stroke or prior dementia, there were 2,437 participants in FHS, of whom 92 developed incident AD dementia, and 743 participants in CHS, of whom 166 developed incident AD dementia (Supplementary Table 2). Participants in CHS were substantially older on average and a higher proportion was female compared to participants in FHS, which contributed to a higher rate of AD dementia in CHS compared to FHS (22.3% versus 3.8%) in the follow-up period (Supplementary Table 2). Contrary to our expectations, the presence of CHIP was associated with a lower subdistribution hazard ratio (SHR) for incident AD dementia in fully adjusted competing risks regression (CRR) models (SHR = 0.69, *P* = 0.13 in CHS; SHR = 0.51, *P* = 0.068 in FHS; SHR = 0.63, *P* = 0.024 in a fixed-effects meta-analysis of the two cohorts), while the effects of age, sex and *APOE* genotype were as expected based on prior studies (Fig. 1a). *APOE* genotype was strongly associated with AD dementia risk in those without CHIP age 60 years or older (*P* = 8.1 × 10^−8^ by log-rank test), but not in CHIP carriers of the same age (*P* = 0.42 by log-rank test) (Fig. 1b), possibly due to smaller sample size. The inclusion of MG-associated germline polymorphisms from AD GWAS did not attenuate the effect of CHIP in these models (Supplementary Table 3).

**Fig. 2:**
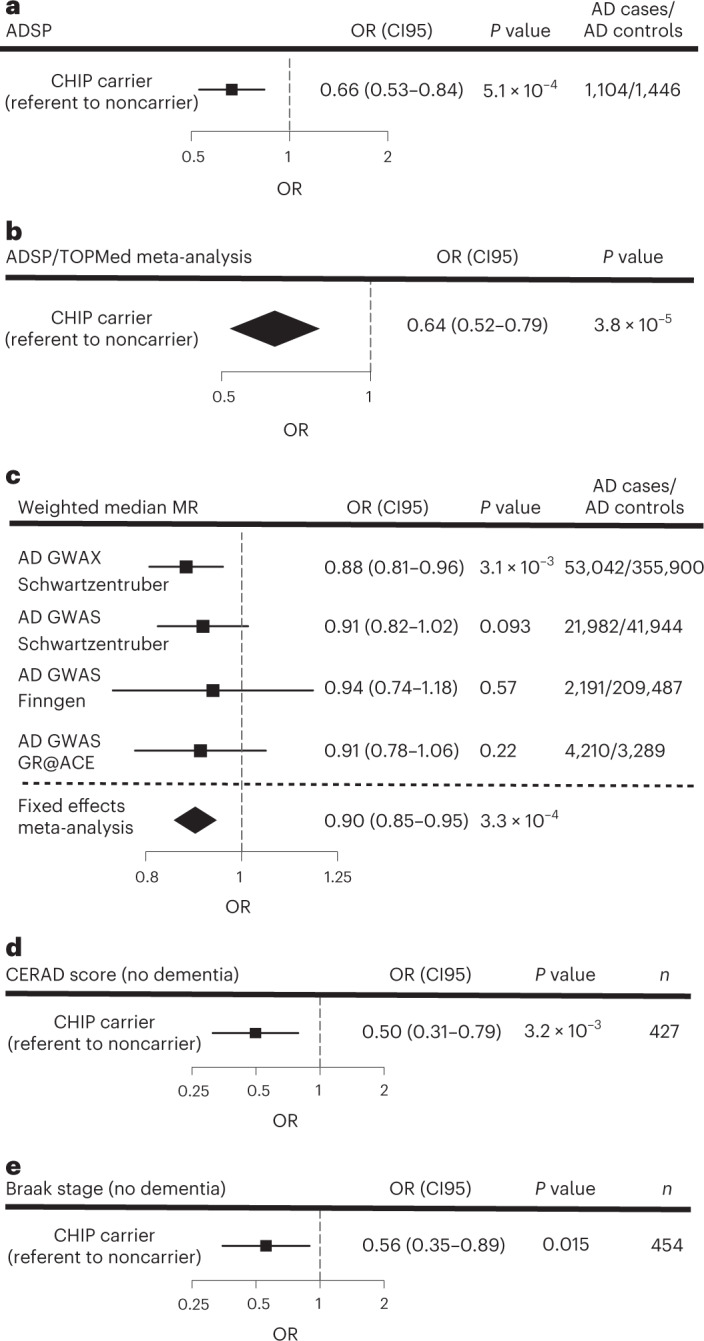
CHIP is associated with protection from AD dementia and AD neuropathologic change (ADNC). **a**. Effect of CHIP on the risk of AD in those with *APOE* ε3ε3 genotype from the ADSP. OR, CI95 and two-sided Wald *P* value were calculated from a logistic regression model that also included age at the time of blood draw and sex as covariates (Supplementary Table 5 for full regression results). People, *n* = 2,550. **b**, Fixed-effect meta-analysis for risk of AD in CHIP carriers using logistic regression in ADSP, FHS and CHS with two-sided Wald *P* value shown. **c**, MR for risk of AD based on genetic risk of CHIP using the weighted median estimator on summary statistics from Schwartzentruber et al.^18^ AD GWAS and GWAX, Finngen AD GWAS and Gr@ACE AD GWAS, which were meta-analyzed using a fixed-effects model. OR, CI95 and two-sided Wald *P* values for risk of AD per 1 log-odds increase in genetic risk of CHIP are shown. The GWAX analysis was one-sample MR (both CHIP and AD effect estimates were derived from UK Biobank data), while the other studies were two-sample MR. People, *n* = 692,045. **d**, Effect of CHIP on the risk of increased CERAD neuritic plaque score in ADSP participants without a dementia diagnosis. OR and CI95 were calculated from an ordinal logistic regression model, which included age at autopsy, *APOE* genotype, sex and CHIP status as covariates. Two-sided *P* values were calculated by comparing the *t*-statistic for each covariate against a standard normal distribution. Full regression results are in Supplementary Table 9. People, *n* = 427. **e**, Effect of CHIP on the risk of increased Braak stage in ADSP participants without a dementia diagnosis. OR and CI95 were calculated from an ordinal logistic regression model, which included age at autopsy, *APOE* genotype, sex and CHIP status as covariates. Two-sided *P* values were calculated by comparing the *t*-statistic for each covariate against a standard normal distribution. Full regression results are in Supplementary Table 9. People, *n* = 454. For all forest plots, the measure of center is the OR and the lines represent the CI95 for the OR.

**Extended Data Fig. 1:**
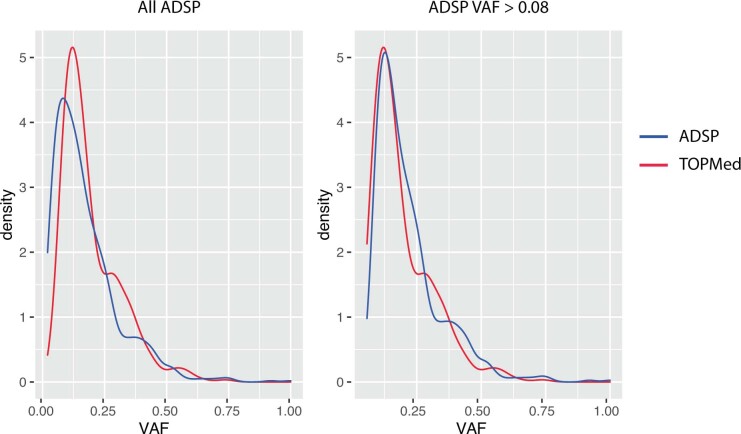
The VAF distribution in TOPMed and ADSP. Density plots of VAF from all CHIP carriers in ADSP (left) or in CHIP carriers with VAF greater than 0.08 (right) compared to VAF distribution from TOPMed CHIP carriers (red) in each plot.

Next, we sought to replicate the association between CHIP and AD dementia in Alzheimer’s Disease Sequencing Project (ADSP), a case-control study with similar criteria for AD diagnosis as TOPMed. Due to a selection process that targeted cases with minimal risk as predicted by known risk factors (age, sex and *APOE* genotype) and targeted controls with the least probability of conversion to AD by age 85 years^16^, only *APOE* ε3ε3 cases and controls were well matched with respect to age and sex (Methods). Therefore, we focused our analysis on the 1,104 individuals with AD dementia and 1,446 individuals without AD dementia who had the *APOE* ε3ε3 genotype (Supplementary Table 4). The sequencing depth in ADSP WES samples (~80×) was higher than that for TOPMed WGS samples (~38×), which resulted in greater sensitivity to detect smaller clones (Extended Data Fig. 1). Clone size, which is approximated by the variant allele fraction (VAF), has previously been shown to be an important predictor of risk for blood cancer^1,17^ and cardiovascular outcomes^8,9^. To directly compare the outcomes of ADSP to TOPMed, both cohorts would need to have a similar distribution of VAF in CHIP carriers. Therefore, we limited the definition of CHIP carriers to those with VAF > 0.08 in ADSP—a cutoff that was empirically chosen because it resulted in a VAF distribution that was nearly identical to TOPMed (Extended Data Fig. 1). We found that CHIP with VAF > 0.08 was associated with lower risk of AD dementia in ADSP using logistic regression (odds ratio (OR) = 0.66, *P* = 5.5 × 10^−4^; Fig. 2a and Supplementary Table 5). In contrast, having VAF ≤ 0.08 had no association with AD dementia (OR = 1.25, *P* = 0.23; Supplementary Table 6). Higher VAF was also significantly associated with protection from AD dementia when modeled as a continuous variable (Supplementary Table 6). The effect of CHIP remained significant when using a VAF cutoff of >0.02 (OR = 0.79, *P* = 0.024; Supplementary Table 5). A meta-analysis of ADSP, CHS and FHS showed that CHIP carriers had a significantly lower risk of AD dementia (OR = 0.64, *P* = 3.8 × 10^−5^; Fig. 2b).

### Mendelian randomization analyses of CHIP and AD

**Extended Data Fig. 2:**
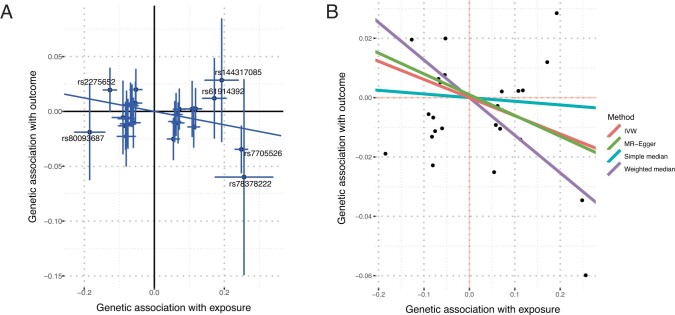
Scatterplot of effect sizes from the Mendelian randomization study. **a**) Regression line for the IVW model is shown. The variants shown are from previously published genome-wide association studies (GWAS) of CHIP as described in Methods and listed in Supplementary Table 7. The measure of center is the beta estimate for each variant and the lines represent the 95 percent confidence interval for the beta estimate. All values were taken from published summary statistics data from CHIP or AD GWAS. **b**) Regression lines for each model are shown using the same data as in A).

MR is a form of causal inference in which inherited genetic polymorphisms known to influence the risk of a particular exposure or trait (in this case CHIP) are assessed for an association with a disease or trait (in this case AD). One- and two-sample MR using 24 CHIP-associated polymorphisms^18^ as the instruments (Supplementary Table 7) found that higher genetic risk of CHIP was associated with reduced odds of AD using the weighted median estimator (OR = 0.90 per 1 log-odds increase in risk of CHIP, *P* = 3.3 × 10^−4^, Fig. 2c). Consistent results were obtained with other MR methods (Supplementary Table 8 and Extended Data Fig. 2). To rule out reverse causation (that having AD causally reduces the risk of CHIP), we also performed two-sample MR using 36 polymorphisms for risk of AD as the instruments^18^ and CHIP as the outcome, which found no evidence of a causal effect in this direction (OR = 0.97 per 1 log-odds increase in risk of AD, *P* = 0.26 using the weighted median estimator).

### Association between CHIP and ADNC

The hallmark neuropathological features of AD, regional accumulation of β-amyloid plaques and tau neurofibrillary tangles, can also be found in some people without a clinical diagnosis of dementia. A neuritic plaque density score developed by the Consortium to Establish a Registry for AD (CERAD)^19^ and Braak stage for neurofibrillary tangle distribution^20^ are commonly used to assess these changes at brain autopsy, with a higher score indicative of more extensive accumulation of pathologic features. A subset of participants in ADSP had a brain autopsy performed after death, which allowed us to test whether CHIP was associated with ADNC in those without dementia (Supplementary Table 9a,b). Here the presence of CHIP was associated with having a lower CERAD score (OR = 0.50, *P* = 3.2 × 10^−3^) and Braak stage (OR = 0.56, *P* = 0.015) using ordinal logistic regression after adjusting for age at death, sex and *APOE* genotype (Fig. 2d,e and Supplementary Table 9c,d).

### Stratified associations between CHIP and AD

**Fig. 3:**
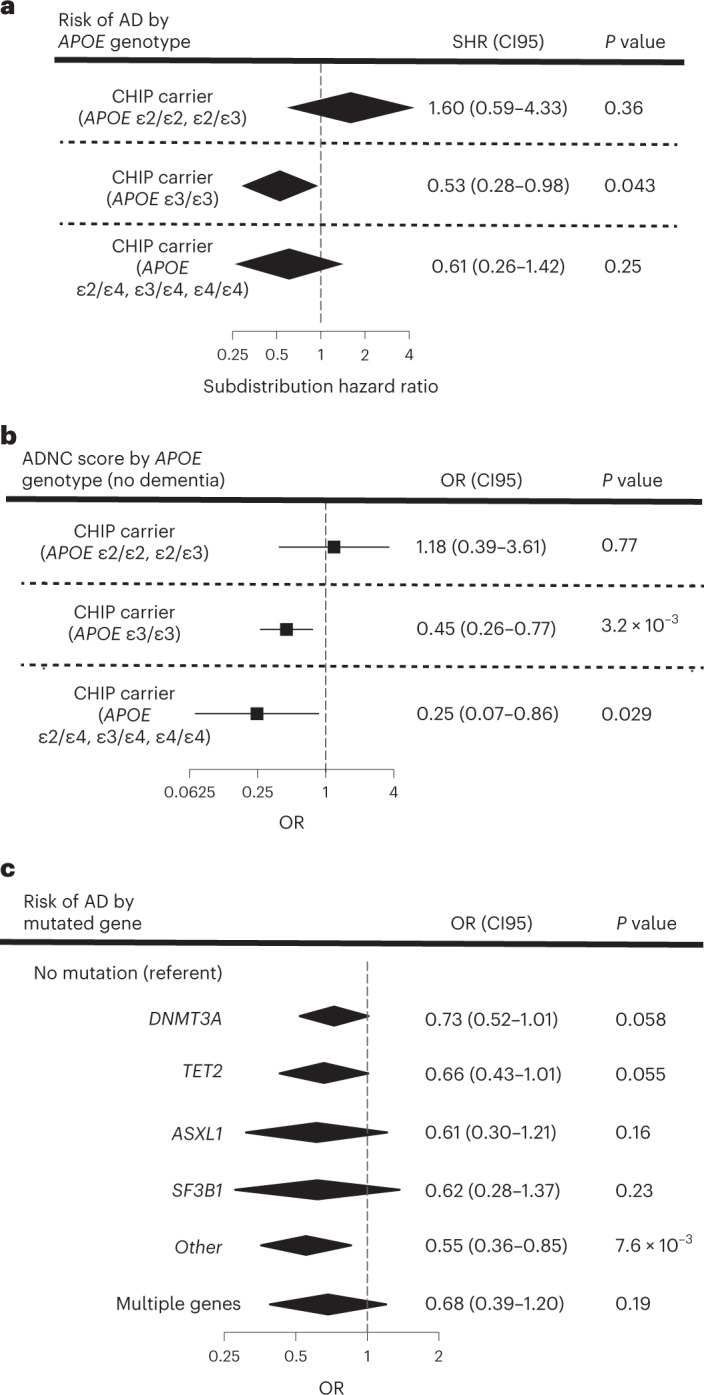
Associations of CHIP to AD by *APOE* genotype and mutated driver gene. **a**, Effect of CHIP on AD dementia risk in participants from CHS and FHS stratified by *APOE* genotype. Participants were binned into those with neutral (*APOE* ε3ε3), low-risk (*APOE* ε2ε2 and ε2ε3) and high-risk (any *APOE* ε4 allele) groups. SHR, 95% confidence intervals (CI95) and two-sided Wald *P* values were calculated for each covariate (age at the time of blood draw for sequencing, sex and CHIP carrier status) from CRR models, and results from FHS and CHS were then meta-analyzed using a fixed-effects model (Supplementary Table 10 for full regression results). Adjustments for multiple comparisons were not performed. People, *n* = 3,180. **b**, Effect of CHIP on ADNC score in cognitively intact participants from ADSP stratified by *APOE* genotype. Participants were binned into those with neutral (*APOE* ε3ε3), low-risk (*APOE* ε2ε2 and ε2ε3) and high-risk (any *APOE* ε4 allele) groups. ORs, CI95 and two-sided Wald *P* values were calculated for a six-point composite score of CERAD and Braak stages and analyzed using an ordinal logistic regression model with age at death, sex and CHIP carrier status as covariates (Supplementary Table 10 for full regression results). Adjustments for multiple comparisons were not performed. People, *n* = 422. **c**, Effect of mutated CHIP gene on AD in participants from CHS, FHS and ADSP. OR, CI95 and two-sided Wald *P* values were calculated for each covariate (age at the time of blood draw for sequencing, sex, cohort and *APOE* genotype) from logistic regression models, and results from the TOPMed cohorts and ADSP were then meta-analyzed using a fixed-effects model (Supplementary Table 12 for full regression results). Adjustments for multiple comparisons were not performed. People, *n* = 5,730. For all forest plots, the measure of center is the SHR or OR and the lines represent the CI95 for the SHR or OR.

*APOE* genotype is the strongest genetic risk factor for AD^21^, with *APOE* ε2 conferring protection from disease and *APOE* ε4 conferring higher risk, compared to the *APOE* ε3 allele (Fig. 1a). In CHS and FHS, the decrement in risk for AD dementia associated with CHIP was of a similar magnitude among people with the *APOE* ε3ε3 genotype or with an *APOE* ε4 allele, but not seen in those with *APOE* ε2ε2 or *APOE* ε2ε3 genotypes (Fig. 3a and Supplementary Tables 10a–c). In ADSP participants without dementia, ADNC was lower in CHIP carriers compared to noncarriers with the *APOE* ε3ε3 genotype or with an *APOE* ε4 allele, but this effect was seen not in those with *APOE* ε2ε2 or *APOE* ε2ε3 genotypes (Fig. 3b). Regression models that examined the interaction of CHIP with *APOE* genotype showed a consistent direction of effect as the stratified models for both AD and ADNC, although the individual interaction terms did not reach statistical significance (Supplementary Tables 9e and 10d). While these results are suggestive, they require confirmation in larger cohorts.

In analyses stratified by sex, there was a similar degree of protection for both male and female CHIP carriers (Supplementary Table 11). We also assessed whether the risk of AD dementia varied based on the specific mutated gene. Of the most commonly mutated genes in CHIP, all were associated with protection from AD dementia to a similar degree (Fig. 3c and Supplementary Table 12).

### Detection of CHIP variants in the human brain

**Fig. 4:**
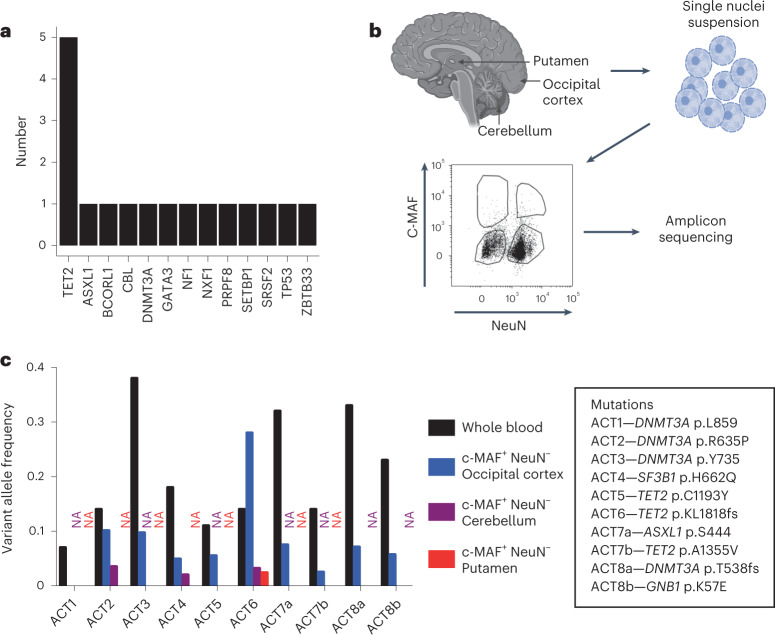
CHIP variants can be found in the MG-enriched fraction of the brain. **a**, Barplot of putative CHIP mutations identified from whole exome sequencing of brain DNA from 1,775 persons in ADSP. Details about the variants identified are in Supplementary Table 13. **b**, Schematic of experimental workflow. Autopsy samples from the occipital cortex, cerebellum and putamen were digested to prepare single nuclei suspensions. Nuclei were then stained and sorted using antibodies to c-MAF^+^ (a marker of myeloid cells) and NeuN^+^ (a marker of neuronal cells), followed by amplicon sequencing for CHIP variants. **c**, Barplot of the VAF of the CHIP variants from eight donors (ACT1 to ACT8). For each sample, the VAF in the blood and in the brain c-MAF^+^ NeuN^−^ population is shown. The occipital cortex was available for all eight donors. A bar for cerebellum or putamen is shown if available, otherwise, NA in the corresponding color designates lack of an available sample (purple for cerebellum and red for putamen). The CHIP mutations present in each participant are reported in the box on the right of the barplot. Details about the samples and the VAF from sorted populations are shown in Supplementary Table 14.

We wondered whether cells bearing CHIP-associated mutations could be found in the brain, a finding that would support a causal association between CHIP and AD risk. We first obtained brain DNA-derived WES data from 1,776 persons in ADSP and assessed for the presence of CHIP-associated variants. Similar to a prior study^22^, we found mutations consistent with CHIP in 17 brain samples (Fig. 4a and Supplementary Table 13), although paired blood samples were not available for this set.

**Extended Data Fig. 3:**
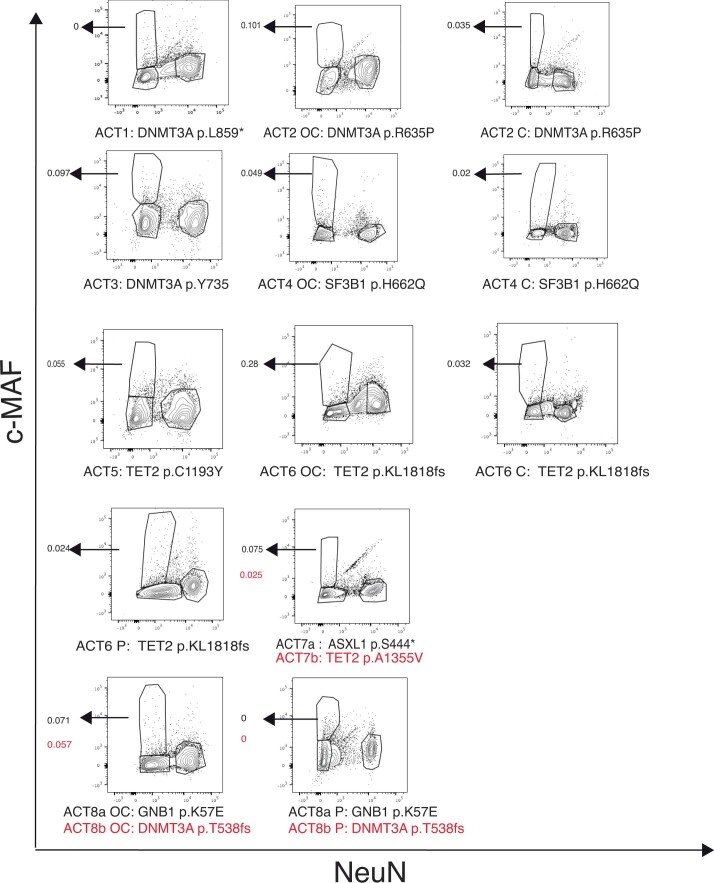
Sorting of nuclei from brains of CHIP carriers. Flow cytometry gating strategy for nuclei sorting for the 8 brain samples using c-MAF and NeuN markers. The arrow points to the c-MAF^+^ NeuN^−^ sorted population and the VAF is indicated for each sample. The brain regions are abbreviated as Ce for Cerebellum, OC for Occipital cortex, and P for putamen.

To test definitively whether blood-derived cells from CHIP carriers were present in the brain, we obtained tissue samples from the occipital lobe, and in some cases putamen or cerebellum, of eight donors from the ACT cohort who were known to have CHIP from blood exome sequencing, as well as four donors without CHIP (Supplementary Table 14). All persons were in their 80s and 10 of 12 were without dementia and had no/low ADNC at the time of death. The eight CHIP carriers had mutations in *DNMT3A, TET2*, *ASXL1*, *SF3B1* and *GNB1* with the highest frequency in *DNMT3A* (four of eight) and *TET2* (three of eight) (Supplementary Table 14). In addition, two of the eight harbored two different CHIP mutations. We digested the frozen brain tissue and isolated intact nuclei, from which we extracted DNA for amplicon sequencing. We detected the same mutations that were present in blood in six of eight unfractionated brains with VAF ranging from 0.004 to 0.02 (Extended Data Fig. 3 and Supplementary Table 14).

**Extended Data Fig. 4:**
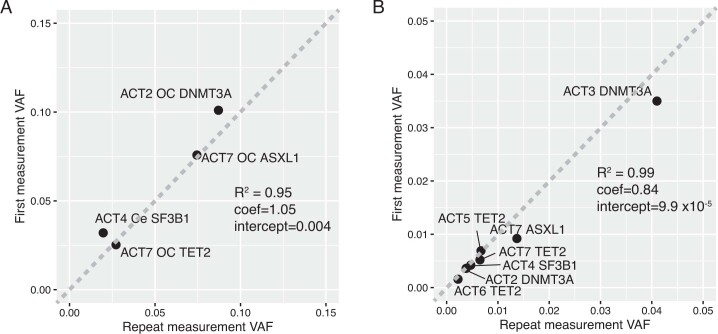
Correlation of repeated VAF measurements from brain. **a**) c-MAF^+^ NeuN^−^ nuclei were sorted from the samples shown and VAF for the CHIP variants was assessed. A repeat sample was sorted to determine the variability in the VAF of sorted populations. **b**) DNA from unsorted occipital cortex nuclei was obtained for the samples shown. VAF of the CHIP variants was determined from two independent sequencing libraries generated for each sample to assess the variability in the VAF across sequencing runs. The y = x line is drawn. R^2^, coefficient, and y-intercept values calculated from linear regression are shown.

We hypothesized that the presence of CHIP mutations represented blood cells, likely myeloid, that had engrafted in the brain. To test this hypothesis, we devised a flow cytometric strategy to enrich myeloid cells and deplete other blood cell types. Because the tissue was not viably cryopreserved, isolation of cells based on the expression of membrane antigens was not possible. Instead, we stained nuclei for the neuronal-specific transcription factor NeuN (encoded by the *RBFOX3* gene) and c-MAF (encoded by the *MAF* gene), a transcription factor highly expressed in mononuclear phagocytes, including monocytes, macrophages, dendritic cells and MG, as well as some neurons and nonhematopoietic glial cells, but not granulocytes or most lymphocytes. We then sorted four populations based on the presence or absence of these markers (Fig. 4b). The CHIP somatic variants were found in the NeuN^−^ c-MAF^+^ population in seven out of eight brains, with a VAF that ranged from 0.02 to 0.28 (representing 4% to 56% of NeuN^−^ c-MAF^+^ nuclei). In contrast, CHIP somatic variants were not detected in the NeuN^+^ c-MAF^−^ neuronal population and were absent or at low levels in the other two populations (Fig. 4c and Extended Data Fig. 3), effectively excluding granulocytes and lymphocytes as the source of the mutations. To determine whether the observed VAF was reproducible across replicates, we repeated the VAF measurement in the NeuN^−^ c-MAF^+^ gate for four mutations and obtained concordant results (Extended Data Fig. 4a).

### Single-nucleus chromatin accessibility profiling of human brains

MG are believed to have little contribution from marrow-derived cells in adulthood^23^, so it would be surprising to find the mutations in this compartment. To better understand the identity of the mutated myeloid cells in the brains of CHIP carriers, we performed single nucleus ATAC-sequencing (snATAC-seq) on brain samples from the four ACT donors without CHIP (ACT9-12), as well as the six donors with detectable CHIP mutations in the unsorted brain (ACT2-7). All ten donors were free of AD and had no/low ADNC (Supplementary Table 14). We analyzed the occipital cortex from all donors to assess the cell state in a single brain region across all samples. We also analyzed the cerebellum and putamen for the ACT6 donor to determine if the infiltration of mutant cells varied by brain region within a single donor. snATAC-seq was performed on unsorted nuclei for each sample, as well as sorted NeuN^−^ c-MAF^+^ nuclei for ACT2, ACT6 and ACT9.

**Fig. 5:**
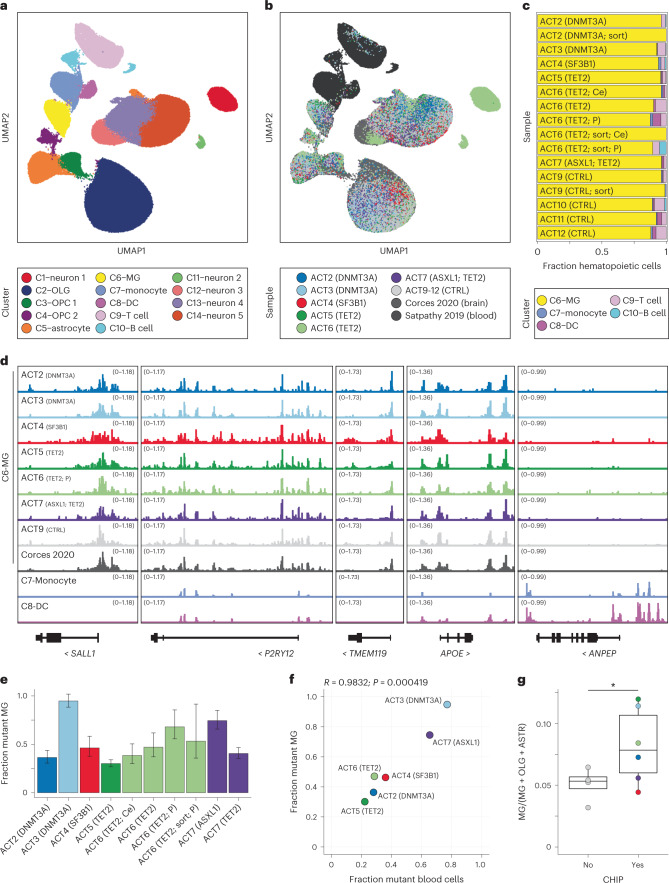
snATAC-seq of brain samples from CHIP carriers reveals that the mutated cells are similar to MG and comprise a large proportion of the microglial pool. **a**, snATAC-seq profiles of 187,754 cells from our dataset, the Corces 2020 adult human brain dataset and the Satpathy 2019 human blood dataset. Each dot represents the snATAC-seq profile of one cell and is colored by its assigned cluster. **b**, snATAC-seq profiles of all cells colored by which donor it originated from. Samples from Corces 2020 and Satpathy 2019 are aggregated and shown in gray and dark gray, respectively. **c**, Cluster composition of hematopoietic cells in each sample in this study. **d**, Pseudo-bulk tracks for selected gene loci. The top eight tracks show snATAC-seq coverage of cells from the indicated sample (or aggregated Corces 2020 samples) within cluster C6–MG. Clusters C7–monocyte and C8–DC are also included for visual reference. **e**, Calculation of the proportion of MG bearing a CHIP mutation in each sample. Error bars depict the simulated 95% confidence interval for percent mutant MG in each sample using *n* = 10^6^ random samples (see Methods for details). **f**, Correspondence between the fraction of mutant blood cells and mutant MG for each donor (*n* = 6). Pearson product-moment correlation coefficient and two-sided *P* value calculated from a *t*-statistic based on the correlation coefficient are shown. **g**, Fraction of MG relative to the total glial pool in occipital cortex samples from CHIP and non-CHIP donors, computed from frequencies of the indicated clusters. Two-sided Wald *P* value = 0.042 by quasibinomial regression of the cell counts of MG, oligodendrocytes and astrocytes. *n* = 10 donors. Box plot shows median, first quartile and third quartile. Box plot whiskers show minimum and maximum values, which are capped at a distance of 1.5× (interquartile range) away from the box. The colors in **d**–**g** represent the sample name as depicted in the box under Fig. 4b. Detailed information for each donor is available in Supplementary Table 14. Samples are unsorted and are taken from the occipital cortex unless otherwise indicated. Ce, cerebellum; P, putamen.

**Extended Data Fig. 5:**
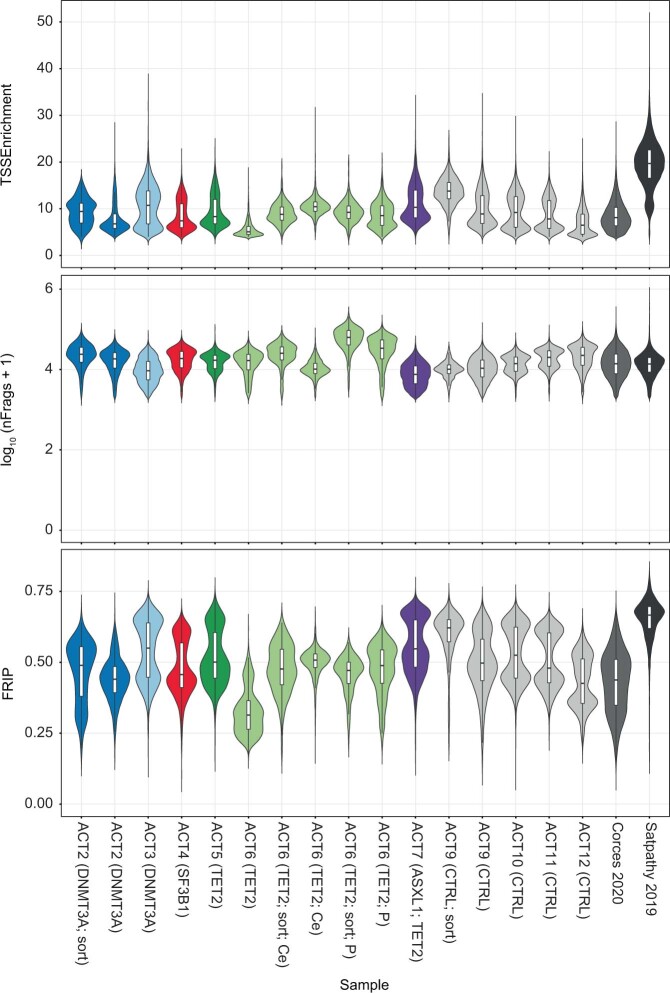
High-quality snATAC-seq libraries from ACT brain samples. Quality control metrics for each nucleus in the indicated scATAC-seq sample. Aggregated Corces 2020 samples and Satpathy 2019 samples are also included. Top: Enrichment of fragments in transcription start sites (TSS). Center: number of fragments. Bottom: fraction of reads in peaks (FRIP) for each nucleus. Unless otherwise indicated, the samples are derived from occipital cortex (OC) and are unsorted. The brain regions are abbreviated as Ce for Cerebellum, OC for Occipital cortex, and P for putamen. The n for each sample is the number of nuclei analyzed in a single experiment for each sample, which is listed in Supplementary Table 15 under column ‘TOTAL’.

**Extended Data Fig. 6:**
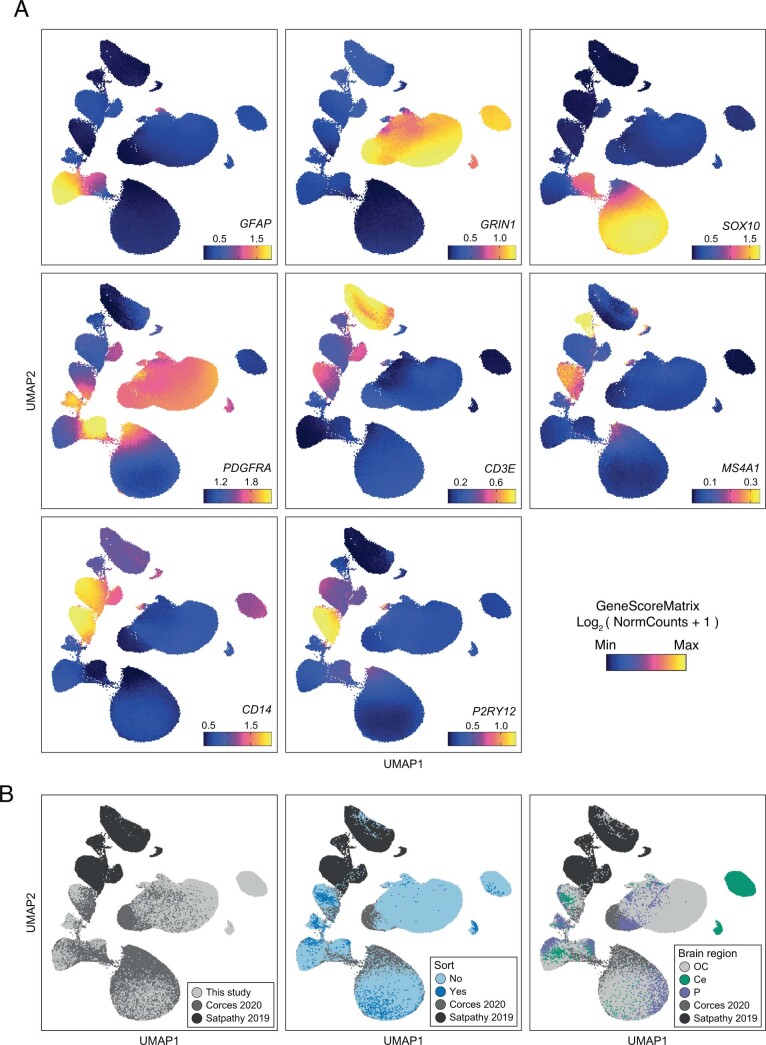
Marker genes and additional data for scATAC-seq clusters. **a**) snATAC-seq profiles of all nuclei colored by accessibility of indicated marker genes (quantified using GeneScore). **b**) snATAC-seq profiles of all nuclei colored by which study they originated from (left), sort status (center), or brain region (right). Brain regions are abbreviated as Ce for Cerebellum, OC for Occipital cortex, and P for putamen.

**Extended Data Fig. 7:**
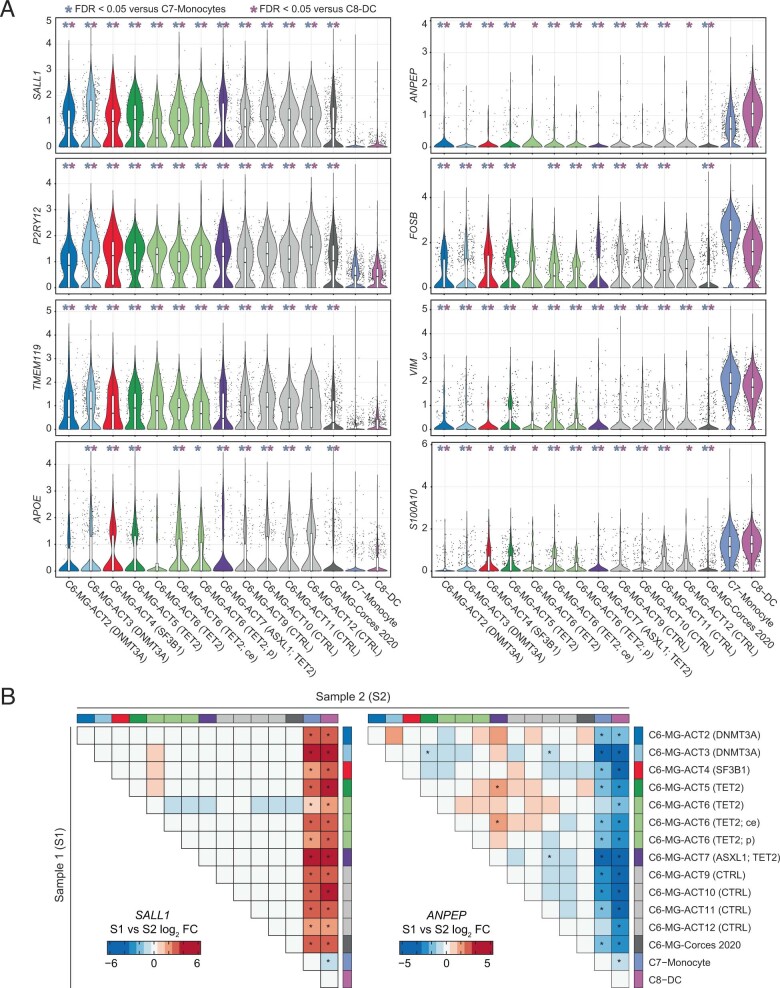
Quantification of gene accessibility in microglia subsets. **a**) For each gene, accessibility in each single nucleus is plotted (quantified via ArchR GeneScore). The n for each brain sample is the number of nuclei in the microglia cluster (C6) analyzed in a single experiment for each sample, which is listed in Supplementary Table 15. n = 7,982 nuclei for C7-monocyte and n = 2,369 nuclei for C8-DC. **b**) Fold change and statistical significance when comparing all pairs of groups for *SALL1* (left) and *ANPEP* (right). (A-B) Within the microglia cluster (C6), cells are split by donor and brain region. Aggregated Corces 2020 microglia, as well as C7-Monocyte and C8-DC clusters, are included for reference. Statistical significance and fold change was computed for all pairs of groups for all GeneScores using the Wilcoxon test. * denotes FDR < 0.05.

**Extended Data Fig. 8:**
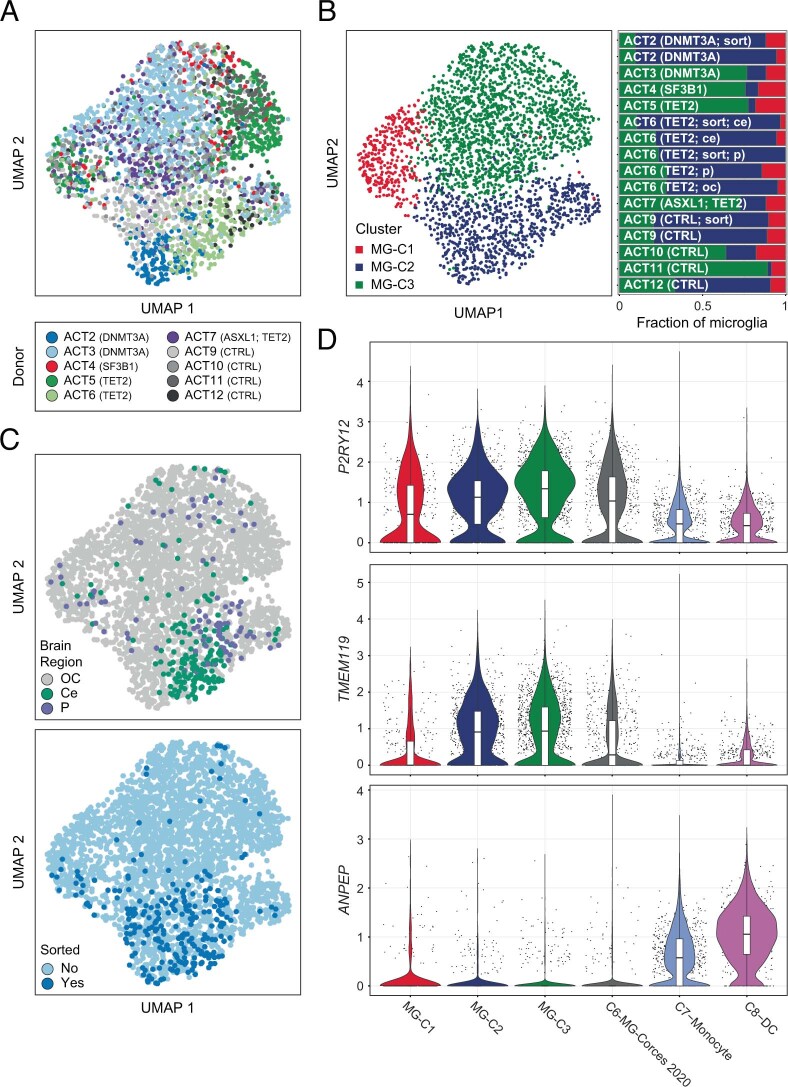
Sub-clustering of cells in our samples within cluster C6-MG. (**a**) snATAC-seq profiles of each nucleus colored by which donor it originated from. (**b**) snATAC-seq profiles colored by sub-cluster (left) and sub-cluster composition of each sample (right). (**c**) snATAC-seq profiles of nuclei colored by brain region (top) or sort status (bottom). (**d**) Quantification of accessibility in each single nucleus for selected marker genes (ArchR GeneScore). Nuclei from our samples are grouped by sub-cluster, and microglia from the Corces 2020 reference dataset, as well as C7 and C8 from Fig. 4, are included for reference.

In total, we recovered high-quality snATAC-seq profiles for 94,367 nuclei (Extended Data Fig. 5). We then aggregated our data with previously published snATAC-seq data from ten samples from the adult human brain,^24^ as well as from circulating immune cells^25^ (additional 93,387 nuclei in total). We identified 14 clusters encompassing the major brain and hematopoietic cell types (Fig. 5a,b), including one cluster that contained previously described MG, as well as myeloid cells from each of our samples (C6; Fig. 5b, Extended Data Fig. 6 and Supplementary Table 15). Small numbers of cells in our brain samples clustered with circulating immune cells, predominantly T cells, and were found in all unsorted brains (mean = 7.2% of total hematopoietic cells, range 3.7–12.5%). There was no enrichment of nonmicroglial hematopoietic cells in the CHIP carriers (mean = 6.5%) compared to controls (mean = 8.7%; Fig. 5c and Supplementary Table 15). For nuclei within C6 grouped by sample, accessible chromatin at MG marker genes (*TMEM119*, *P2RY12*, *SALL1* and *APOE*) in each of our samples was visually and quantitatively similar to the reference MG but distinct from profiles of blood monocytes or dendritic cells (Fig. 5d and Extended Data Fig. 7). Furthermore, the C6 tracks in all brain samples had low accessibility at *ANPEP* (CD13), *FOSB*, *VIM* and *S100A10*, in contrast to monocytes or DCs. To further investigate heterogeneity within C6, we reclustered cells from our samples within C6 (Extended Data Fig. 8). We identified three subclusters, all of which exhibited accessibility profiles consistent with MG, but we did not observe consistent differences in subcluster proportions between CHIP and non-CHIP samples (Extended Data Fig. 8). In total, these results indicate that the cells in C6 exhibit aggregate similarity to MG and are distinct from monocytes (C7) and dendritic cells (C8). Furthermore, the C6 cells in CHIP carriers were similar to C6 cells in noncarriers in several different analyses.

We next used the snATAC-seq data to estimate the proportion of mutated MG in each sample. We determined that the percentage of MG ranged from 7.6% to 25% in the sorted samples, representing an 11.5-fold to 34.5-fold enrichment for MG in the occipital cortex and cerebellum (Fig. 5e and Supplementary Table 16). However, this indicated that there were still large numbers of contaminating non-MG cells in the NeuN^−^ c-MAF^+^ gate. To better estimate the percentage of mutated MG, we first assessed the VAF for the CHIP variant in each unsorted brain sample. We confirmed that VAF estimates from unsorted nuclei were consistent across replicates of the same sample (Extended Data Fig. 4b). We also determined that the VAF estimates were unlikely to be inflated by artifactual variant reads because amplicon sequencing of a brain sample from a donor without CHIP did not detect any mutant alleles (Supplementary Table 14). Because these are heterozygous mutations, multiplying the VAF by two gives an estimate of the percentage of mutated cells in the sample. We conservatively assumed that all nonmicroglial hematopoietic cells were mutant and subtracted the number of these cells from the total mutant cell count (Supplementary Table 15 and Methods). Having excluded the contribution of all nonmicroglial hematopoietic cells, we reasoned that we could divide the number of remaining mutant cells by the total number of MG in each unsorted sample to estimate the percentage of mutant MG. Overall, we observed a range of mutant MG from 30% to 95% in the six CHIP samples (Fig. 5e and Supplementary Table 16). For the ACT6 donor, 68%, 38% and 47% of putamen, cerebellar and occipital cortex MG harbored the *TET2* mutation, respectively, suggesting that multiple brain regions in CHIP carriers are likely to be infiltrated by marrow-derived myeloid cells that resemble MG. We observed a very strong correlation between the proportion of mutated cells in peripheral blood and in occipital cortex MG from the six CHIP donors (*R*^2^ = 96%, Fig. 5f). This suggests a linear relationship between the size of the mutant clone in the periphery and infiltration of the brain and may contribute to the observed association between higher peripheral blood VAF and protection from AD dementia (Supplementary Table 6).

Finally, as CHIP mutations are known to increase the fitness of HSCs and myeloid progenitors^5^, we looked for evidence of enhanced fitness of mutant MG in these samples. The mean proportion of MG amongst all glia was 5.1% in the four control occipital cortex samples (Fig. 5g), but this proportion was increased to 8.2% in the six occipital cortex samples from CHIP carriers (*P* = 0.042). If confirmed in larger studies, these results may suggest a possible role for CHIP mutations in increasing MG number via enhanced homing, survival or proliferation of marrow-derived MG or their precursors in the brain.

## Discussion

We provide here several lines of evidence supporting the role of mutant, marrow-derived cells in protecting against the risk of AD. First, CHIP is associated with protection from AD dementia in multiple cohorts, an effect that could not be attributed to survival bias. Second, MR analyses were consistent with a causal role for CHIP in reducing AD risk. Third, CHIP was associated with lower levels of neuritic plaques and neurofibrillary tangles in those without dementia, indicating a possible modulating effect of CHIP on the underlying pathophysiology of AD. Fourth, consistent with this hypothesis, we also provided preliminary evidence of substantial infiltration of the brain by marrow-derived mutant cells, which adopted a microglial-like phenotype. The degree of protection from AD dementia seen in CHIP carriers is similar to carrying an *APOE* ε2 allele, which is the most protective common inherited variant for AD^26^.

One of the more surprising findings in our study is the high proportion of mutant MG-like cells observed in the brains of CHIP carriers. This was demonstrated by amplicon sequencing of nuclei from both sorted and unsorted brain fractions, although the estimated fraction of mutant cells is likely more reliable from the unsorted brain. Although there is some uncertainty in the estimates due to known or unknown causes of measurement error, we found a very strong correlation between the mutant cell fraction in blood and MG, arguing against imprecision in the estimates due to stochastic variation from sample preparation or sequencing. We were also able to rule out contamination by circulating blood cells as an explanation for this finding, as ~88% to 96% of the hematopoietic cells in the brain samples clustered as MG and were distinct from other immune cells. Nonetheless, our study has limitations. We did not find differences in chromatin accessibility between the MG from CHIP carriers compared to noncarriers that could explain the protection from AD. This may be due to limited sample size, mutational heterogeneity or the fact that we only assessed MG from people without AD or significant ADNC. It is possible that differences in microglial phenotypes due to CHIP are not evident at steady-state, but only after perturbation, as has been observed in other contexts^8,11,27,28^. Therefore, while the data presented here are suggestive, we cannot currently conclude that the mechanism of protection from AD seen in CHIP carriers is due to these infiltrating cells. Identifying biologically meaningful differences between mutant and wild-type MG would strengthen the hypothesis that mutant cells residing in the brain have a causal role in protection from AD. Functional studies of mutant MG are likely to be informative, as are technologies that are able to obtain both genotype and transcriptome data from single cells within the same sample^29,30^.

There is a growing body of evidence from mice that bone marrow-derived myeloid cells can enter the brain during homeostasis^31,32^. Our study demonstrates that this may be true in humans as well. Marrow-derived cells may have an ameliorative effect on neurodegenerative phenotypes in mice^33,34^, possibly due to their superior phagocytic capacity^35^. GWAS have strongly implicated microglial genes involved in ligand recognition and phagocytosis, such as *APOE*, *TREM2*, *CD33* and the MS4A cluster, in the biology of AD^36^. We hypothesize that reduced phagocytic capacity of the endogenous microglial system during aging elevates the risk of AD, but that this is rescued in CHIP carriers, possibly due to the influx of peripherally derived myeloid cells into the brain parenchyma, which are able to outcompete endogenous MG. The observation that the effect sizes were similar with several different driver genes suggests that there could be shared mechanisms leading to protection from AD across several different mutations. For example, there may be convergent gene expression patterns in innate immune cells or a common effect of the mutations on MG survival or proliferation, similar to what has been observed in other contexts^12,37^. Along these lines, we provide preliminary evidence of a quantitative increase in the fraction of MG in the brains of CHIP carriers. We predict that more detailed characterization of phenotypic differences between mutant and wild-type MG may provide insights into slowing the progression of AD.

## Methods

### Study population

#### FHS

The FHS is a single-site, prospective and population-based study that has followed participants from the town of Framingham, MA to investigate risk factors for cardiovascular diseases. The population of Framingham was almost entirely white at the beginning of the study. The FHS comprises three generations of participants. The first generation (Original cohort/Gen1) (ref. ^38^), followed since 1948, enrolled 5,209 male and female participants who comprised two-thirds of the adult population then residing in Framingham, MA, USA. Survivors continue to receive biennial examinations. The second generation (Offspring cohort/Gen2) (ref. ^39^), followed since 1971, comprised 5,124 offspring of Gen1 and spouses of the offspring (including 3,514 biological offspring) who attended examinations every 4–8 years. The third generation (Gen3) (ref. ^40^), enrolled in 2002, included 4,095 children from the largest offspring families who attended three examinations 4 years apart. All cohorts continue under active surveillance for cardiovascular events, stroke and dementia. The FHS was approved by the Institutional Review Board of the Boston University Medical Center. All study participants provided written informed consent at each examination.

A total of 4,195 samples were sequenced as part of the TOPMed project Freeze 6 release as previously described^9^. The selection of participants for sequencing was mostly a random selection of those with available DNA but also included some related individuals for family studies. After the exclusion of participants with coronary heart disease, ischemic stroke or with missing information on age at blood draw or AD diagnosis, a total of 2,437 persons remained for this analysis. Adjudication for Alzheimer’s phenotypes was done by a committee^41^, comprising at least two neurologists and a neuropsychologist. Multiple types of information were used to evaluate participants with suspected dementia, including neurologic and neuropsychological assessments, a telephone interview with a family member or caregiver, medical records, imaging studies and autopsy data when available. AD was diagnosed when participants met the criteria of the NINCDS and the ADRDA for definite, probable or possible AD.

#### CHS

The CHS is a prospective, multi-ethnic, longitudinal study of risk factors for coronary heart disease and stroke in people aged 65 and older. A total of 2,840 samples were sequenced as part of the TOPMed project as previously described^9^. The samples selected for WGS as part of TOPMed were heavily oversampled for cardiovascular disease cases. After exclusion of participants with coronary heart disease or stroke, or with missing information on age at blood draw or AD diagnosis, a total of 743 persons remained for this analysis. AD was diagnosed as probable and possible following the NINCDS–ADRDA criteria in 1997–98 and 2002–03 (ref. ^42^). All CHS participants provided informed consent, and the study was approved by the Institutional Review Board of the University of Washington.

Each study in TOPMed received institutional certification before deposition in dbGaP, which certified that all relevant institutional ethics committees approved the individual studies and that the genomic and phenotypic data submission was compliant with all relevant ethical regulations. Secondary analysis of the dbGaP data in this manuscript was approved by the Stanford University Institutional Review Board, and this work is compliant with all relevant ethical regulations.

#### ADSP

The ADSP is a collaborative effort of the National Institutes of Aging, the NHGRI and the Alzheimer’s community to understand the genetic basis of AD^16^. The whole exome sequencing (WES) set of ADSP was a case-control design where cases met NINCDS–ADRDA criteria for possible, probable or definite AD had documented age at onset or age at death and *APOE* genotyping. A case-control selection strategy was chosen that targeted cases with minimal risk as predicted by known risk factors (age, sex and *APOE*) and targeted controls with the least probability of conversion to AD by age 85 years. A total of 5,096 cases and 4,965 controls from 24 cohorts were chosen for WES. This selection strategy was chosen to maximize the power to detect germline variant associations. Written informed consent was obtained from all human participants in each of the studies that contributed to ADSP in our analysis, which are listed below along with the name of the institutional review board that approved the study. Secondary analysis of the dbGaP data in this manuscript was approved by the Partners Healthcare and Stanford University Institutional Review Boards, and this work is compliant with all relevant ethical regulations.

ACT—University of Washington IRB

National Institute on Aging ADC—39 centers contributed to this data and IRBs at each institution approved the study

CHAP—Rush University Medical Center IRB

EFIGA—Columbia University IRB

NIA-LOAD—Columbia University IRB

MAP—Rush University Medical Center IRB

NCRAD—Indiana University IRB

ROS—Rush University Medical Center IRB

TARC—Baylor College of Medicine, Texas Tech University Health Sciences Center, University of North Texas Health Science Center, The University of Texas Health Sciences Center at San Antonio, The University of Texas Southwestern Medical Center and Texas A & M Health Science Center IRBs

### Variant calling and annotation

WGS of TOPMed samples^15^ and WES of ADSP samples^15^ were previously performed. FASTQ files were aligned to reference genome hg38 for TOPMed WGS and hg19 for ADSP WES and the resulting BAM files were passed through Mutect/Indelocator (ADSP) or Mutect2 (TOPMed) pipelines to identify putative variants^15^. The Mutect/Mutect2 pipelines were run at default settings in Tumor-Normal mode, with one person from each cohort known not to have CHIP used as the ‘normal’ sample. These algorithms excluded variants that had characteristics of common artifacts, such as oxoguanine artifact, end-of-read artifact and PCR artifact (strand bias). Common polymorphisms present in germline databases were also excluded. Rare error modes were excluded by using a Panel of Normals compiled from 1,000 persons without CHIP in the same sequencing centers. Output from the Mutect/Mutect2 pipelines were then annotated for known CHIP variants in 73 genes from a curated whitelist (Supplementary Table 1).

### Statistical analysis plan

#### TOPMed

We wished to test for an association of AD dementia to CHIP. We hypothesized that CHIP carriers would have an increased risk of AD dementia based on prior data that CHIP carriers have more inflammation in innate immune cells^11^ and that enhanced inflammasome activation was associated with worsened AD phenotypes in mice^43^.

For the discovery set, we used the two cohorts in TOPMed with available data on incident AD diagnoses, FHS and CHS. The CHS sample was heavily oversampled for those with cardiovascular diseases, especially coronary heart disease (CHD) and stroke (1,838 of 2,840 participants had these conditions). CHIP is known to be associated with atherosclerotic cardiovascular disease^11^. As systemic atherosclerosis is a risk factor for vascular dementia, which can mimic AD dementia symptoms, we wished to exclude anyone with these conditions to prevent confounding. To do this, we excluded anyone with an event type of myocardial infarction (MI), stroke, angioplasty, coronary artery bypass surgery, silent MI or death due to coronary heart disease using CHS event codes 1, 3, 7, 8, 10 and 11 (https://www.ncbi.nlm.nih.gov/projects/gap/cgi-bin/variable.cgi?study_id=phs000287.v2.p1&phv=100824&phd=2793&pha=3548&pht=1466&phvf=&phdf=&phaf=&phtf=&dssp=1&consent=&temp=1) and excluded those selected on the basis of CHD, stroke or ‘other’ sampling group codes 3, 4, 5 and 6 (https://www.ncbi.nlm.nih.gov/projects/gap/cgi-bin/variable.cgi?study_id=phs001368.v2.p2&phv=377683&phd=8024&pha=&pht=7957&phvf=&phdf=&phaf=&phtf=&dssp=1&consent=&temp=1). For FHS, we also excluded anyone with codes for coronary heart disease or ischemic stroke. For both cohorts, we also excluded anyone with prior dementia. The final study sample for CHS was 743 people, 491 of these were female. The median age was 72 years at the time of blood draw for WGS. There were 123 people with ε2ε2 or ε2ε3 genotype, 26 people with ε2ε4 genotype, 424 people with ε3ε4 genotype, 162 people with ε3ε4 genotype, and 8 people with ε4ε4 genotype at APOE. The final study sample for FHS was 2,437 people, 1,385 of these were female. The median age was 61 years at the time of blood draw for WGS. There were 310 people with ε2ε2 or ε2ε3 genotype, 46 people with ε2ε4 genotype, 1,584 people with ε3ε3 genotype, 458 people with ε3ε4 genotype and 39 people with ε4ε4 genotype at *APOE*.

FHS and CHS are both prospective studies with information on incident AD diagnosis. We, therefore, used time-to-event regression models to test for an association of CHIP to incident AD dementia in both cohorts. To exclude confounding due to survivorship bias, we performed the analysis using CRR, with death as the competing risk. After excluding those without information on AD diagnosis, there were 2,437 persons in FHS and 620 persons in CHS. Other variables included in these models were age at blood draw used for sequencing, *APOE* genotype, and sex for both cohorts and in addition, self-reported race and study site for CHS. For FHS, some participants were selected as part of family studies, which could potentially lead to biased estimates in the regression models due to correlated genetic or environmental factors. To control for this possibility, we also included family as a cluster variable in the CRR model for FHS using the R package crrSC (https://cran.r-project.org/web/packages/crrSC/index.html). The results from both cohorts were then meta-analyzed using a fixed-effects meta-analysis. The R packages meta (https://cran.r-project.org/web/packages/meta/index.html) and cmprsk (https://cran.r-project.org/web/packages/cmprsk/index.html) were used to perform the meta-analysis and CRR, respectively. For both risk of AD and death, visual examination of a plot of the Schoenfeld residuals revealed that the proportional hazards assumption was met for each covariate and visual examination of a plot of the Martingale residuals for age revealed that the linearity assumption was met.

#### ADSP

Having demonstrated a surprising inverse association between CHIP and AD dementia in the discovery set, we wished to replicate the finding. For this, we used the ADSP data. As described above, the selection strategy for ADSP was chosen to maximize the power to detect germline variant associations. As CHIP is strongly associated with age, and to a lesser extent sex, this study design can lead to selection biases that confound a CHIP analysis. These biases resulted in imbalanced distributions for cases and controls based on these variables. This is illustrated by the distribution of AD cases and controls amongst *APOE* ε4 carriers, where AD cases were strongly enriched for younger females and controls were preferentially older males. This age and sex imbalance did not occur for *APOE* ε3/ε3 carriers, presumably because the overall neutral effect of ε3/ε3 genotype led to relaxed selection based on age and sex. Furthermore, 1,776 samples were sequenced from the brain, not blood, DNA. We limited our CHIP/AD association analysis to those samples where DNA was derived from blood and where the age at blood draw used for sequencing was known. In most cases, the AD diagnosis was made before the blood draw; however, the diagnosis was usually within 5 years of the time of blood sampling for both prevalent and incident cases. To assess whether there was an association between CHIP and *APOE* genotype, which could confound our results, we determined the prevalence of *APOE* genotypes stratified by CHIP status in TOPMed. Among 4,276 people without CHIP in TOPMed, 2,688 (62.9%) were *APOE* ε3ε3, 600 (14.0%) were ε2ε2 or ε2ε3 and 988 (23.1%) were ε2e4, ε3ε4 or ε4ε4. Among 485 people with CHIP in TOPMed, 321 (66.2%) were *APOE* e3e3, 59 (12.2%) were ε2ε2 or ε2ε3, and 105 (21.6%) were ε2ε4, ε3ε4 or ε4ε4. Thus, CHIP is not associated with *APOE* genotype.

A second major difference between ADSP and TOPMed is the use of higher-depth WES in ADSP, compared to lower-depth WGS in TOPMed. To perform a power calculation for the replication study in ADSP, we had to ensure the VAF was comparable between ADSP and TOPMed for two reasons. First, the sensitivity to detect CHIP is linked to the sequencing depth, therefore the prevalence of CHIP was higher in ADSP. Second, the associations for previously studied health outcomes related to CHIP are dependent on clone size, with small clones having less of an effect size. We empirically determined that a cutoff of VAF at 0.08 gave a nearly identical VAF distribution for CHIP clones in ADSP as compared to TOPMed (Extended Data Fig. 1).

After excluding those without blood DNA or known age at blood draw and further limiting to *APOE* ε3ε3 carriers, we had 1,104 AD cases and 1,446 controls who were well matched by age. The median age was 81 years at the time of blood draw for WES and there were 1,458 females. We then used the powerMediation (https://cran.r-project.org/web/packages/powerMediation/index.html) package in R to perform a power calculation for varying effect sizes of CHIP at an alpha of 0.1. For an OR of 0.6 (similar to the hazard ratio for CHIP obtained from TOPMed), the power was 1. For an OR of 0.8, the power was 0.96. For an OR of 0.9, the power was 0.50. Thus, we were well-powered for the replication analysis in ADSP. We used logistic regression to assess the association between CHIP and AD in ADSP, with age at blood draw and sex as other explanatory variables in the model. For privacy concerns, those age 90 or older did not have an exact age available on dbGaP, and were considered to be age 90 for the purposes of this analysis.

We further assessed whether smaller clones were associated with AD dementia in two ways. First, we performed a logistic regression for AD where CHIP status was modeled as a three-factor variable (no CHIP, CHIP with VAF ≤ 0.08 or CHIP with VAF > 0.08). Second, we performed a logistic regression for AD dementia where VAF was included as a continuous variable using only CHIP carriers.

A fixed-effect meta-analysis for the risk of AD in CHS, FHS and ADSP was performed using logistic regression models for each cohort with age at blood draw, sex, APOE genotype and CHIP carrier status as covariates.

We wished to test whether CHIP status was associated with AD-related pathologic changes in people without clinical dementia symptoms. For a subset of participants in ADSP who died and donated their brains for research, a neuritic plaque score based on the CERAD criteria and Braak stage was assessed. For this analysis, we used all *APOE* genotypes and limited the analysis to those with available age at autopsy. Information on CERAD score was obtained from the NACC/ADRC and the ACT cohort. Information on Braak stage was available from NACC/ADRC, ACT, FHS and the GD cohorts. For all cohorts, anyone with a clinical dementia diagnosis was excluded. For NACC/ADRC and ACT, we also excluded anyone with mild cognitive impairment. A total of 427 persons had CERAD neuritic plaque scores and 454 persons had Braak stages available for this analysis after these exclusions. We performed ordinal logistic regression for CERAD score (0–3) and Braak stage (grouped as 0/I/II (1), III/IV (2) and V/VI (3)) using the PoLR function in the MASS package in R (https://cran.r-project.org/web/packages/MASS/index.html). Explanatory variables included CHIP, age at death, sex and *APOE* genotype. The *t* values from the ordinal logistic model were used to calculate *P* values for each of the covariates using a standard normal distribution. For the analysis of CHIP and ADNC stratified by *APOE* genotype, we created a composite score of CERAD amyloid burden and Braak tau spread by adding the two scores together, yielding a total score that ranged from 1 to 6. The composite score was then analyzed using ordinal logistic regression adjusted for age at death, sex and CHIP status.

#### Sex-specific analyses

We assessed whether there were sex-specific effects for CHIP by performing analyses for males and females separately in CHS, FHS and ADSP. Sex was self-reported in these cohorts. We used logistic regression models with AD as the outcome variable and age at blood draw, CHIP status, APOE genotype (for CHS and FHS), race (for CHS) and study site (for CHS) as explanatory variables. Family was used as a cluster variable in FHS. Results were then meta-analyzed using fixed-effects models.

#### Mendelian randomization

To perform two-sample MR, we used the 27 independent genome-wide significant hits from ref. ^44^ as the instrumental variables for CHIP exposure (Supplementary Table 7) and summary statistics from several AD association studies for the outcome. Of the 27 CHIP GWAS loci, 24 were available in the AD GWAS summary statistics from ref. ^18^, which provided summary statistics from a large AD GWAS/GWAX meta-analysis. The AD GWAS included ~22,000 AD cases and ~42,000 controls from ref. ^45^, while the GWAX contained ~53,000 AD-by-proxy cases and ~378,000 controls from UK Biobank. We further obtained summary statistics from an AD GWAS from Finngen release 5 (case definition: Dementia in AD F5) comprising 2,191 cases and 209,487 controls and for an AD GWAS from Gr@ACE^46^ comprising 4,210 AD cases and 3,289 controls. The β values and standard errors for the 24 variants from the CHIP and AD GWAS summary statistics were then used to perform weighted median MR, inverse-variance weighted MR using a multiplicative random effects model, mode-based estimation MR and MR-Egger MR using the MendelianRandomization package (https://cran.r-project.org/web/packages/MendelianRandomization/index.html). We also performed analyses using MR-PRESSO (https://github.com/rondolab/MR-PRESSO) and MR-RAPS (https://github.com/qingyuanzhao/mr.raps). Weighted median MR was chosen as the primary analysis due to its ability to provide robust estimates under several scenarios; for example, the estimate would be reliable even if up to 50% of the instrumental variable weight was invalid due to horizontal pleiotropy^47^ (https://wellcomeopenresearch.org/articles/4-186/v2). The results from each GWAS or GWAX were meta-analyzed using fixed-effects meta-analysis in the meta package in R.

The assumptions for the validity of MR analyses are relevance, independence and exclusion restriction of the independent variables (IVs)^48^. We explain why the particular IVs chosen for our study satisfy these assumptions below.

Relevance: We selected as our instrumental variables 24 independent variants recently identified as associated with CHIP in a large GWAS at *P* < 5 × 10^−8^ (Supplementary Table 7). Given the genome-wide significance threshold for inclusion, we consider these to be strong instruments. We estimated the narrow-sense heritability (*h*^2^) for CHIP to be 9.3% using the sum of the scaled marginal effects of these 24 variants. CHIP has moderate heritability relative to other traits (https://nealelab.github.io/UKBB_ldsc/index.html).

Independence: This assumption presupposes that the CHIP IVs are not associated with unmeasured confounders. While this cannot be assessed directly, the detection of associations between IVs and other measured covariates might suggest violations of this assumption. Well-established risk factors for AD that were included as covariates in our observational study were female sex and *APOE* genotype. Clearly, our selected germline IVs are independent of sex and other unlinked genetic variants. See also below for an analysis of the association between the genetic risk of CHIP and hypertension, another potential confounder that could violate the independence assumption. Finally, we obtained similar effect estimates in all of the cohorts we examined, arguing against cohort-specific confounding.

Exclusion restriction: A final assumption is that the CHIP IVs influence AD risk only through CHIP and not in other ways (no horizontal pleiotropy). Like the independence assumption, this is difficult to test directly. To address this, we used several different approaches. First, we used MR methods that are robust to pleiotropy including weighted median (primary analysis), weighted mode, MR-RAPS and MR-PRESSO (Supplementary Table 8). All methods provided similar effect estimates. We further used the MR-PRESSO global test^49^ to assess for horizontal pleiotropy and found no significant *P* values in the four studies used in the MR meta-analysis (UKB GWAX *P* = 0.07, UKB GWAS *P* = 0.20, FinnGen *P* = 0.84 and GR@ACE *P* = 0.91, all *P* values unadjusted for multiple comparisons). Second, we assessed whether any of the proximal genes to CHIP IVs had plausible connections to AD via other mechanisms. None of these genes have an established biological link to AD, but some are thought to be relevant to cancers generally, including solid tumors (*TERT*, *PARP1*, *ATM*, *TP53*, *BCL2L1* and *SETBP1*). This is relevant because previous observational studies have found inverse associations between cancer and AD, but the mechanism for this is unknown^50^. It is unlikely that tumors of solid organs could directly influence the biology of AD. We speculate that the association between cancer and AD could instead be due to carriers of solid tumors having enrichment of genetic variants that also increase the risk of CHIP, which is an area for future investigation. Along these lines, a recent paper found that the genetic liability of CHIP was associated with an increased risk of lung, prostate, ovarian, oral and endometrial cancers^7^. Finally, we noticed that there were multiple shared loci in the CHIP GWAS and a recent GWAS for hypertension^51^. As mid-life hypertension has been reported to be associated with an increased risk of AD in observational studies^52^, we wondered whether the association between CHIP and AD was mediated by hypertension. We, therefore, performed MR using CHIP as the exposure and systolic blood pressure as the outcome. We found that there was a significant effect in some methods, but not others (IVW, effect = 0.144, *P* = 0.62; weighted median, effect = 0.666, *P* = 6.3 × 10^−5^; MR-PRESSO, effect = 0.144, *P* = 0.63; MR-RAPS, effect = 0.173, *P* = 0.014). However, the directionality of effect in all models indicated that increased genetic risk of CHIP was associated with higher blood pressure. Given that higher blood pressure is a risk factor for AD, this does not explain the inverse association between CHIP and AD seen in our MR analysis and observational study.

To assess the reverse association, we used 36 genome-wide significant variants for AD risk from Schwartzentruber et al.^18^ as the instrumental variables and the summary statistics from the CHIP GWAS from ref. ^9^ in two-sample weighted median MR analysis.

### Nuclei isolation from human postmortem brain tissue

ACT is a longitudinal, community-based observational study of brain aging in participants older than 65 randomly sampled from the Group Health Cooperative (now Kaiser Permanente Washington), a health management organization in King County, Washington. A subset of participants in the study donate their brains for research upon death, and a comprehensive neuropathological exam is performed to assess for AD and related neurodegenerative disease pathologies^53^. For decedents with postmortem intervals of less than 8 h, a rapid autopsy is performed in which numerous samples from multiple brain regions are taken from one hemisphere and flash-frozen in liquid nitrogen. Consent for brain donation was obtained from each donor and the study was approved by the University of Washington Institutional Review Board and by the Kaiser Permanente Washington Institutional Review Board.

For this analysis, we obtained occipital cortex samples from 12 ACT brain donors (eight CHIP carriers and four noncarriers). Three of these also had a frozen sample from the cerebellum available, and two had a frozen sample from the putamen. The eight CHIP carriers represented all donors known to have CHIP and with autopsy specimens available. The four without CHIP represented a random selection of the study cohort. Of the 12 donors, two had AD dementia, seven were female, ten were APOE ε3ε3, two were APOE ε3ε4 and the median age at autopsy was 90.5 years. For nuclei isolation, we performed and adapted the protocol from ref. ^54^. Briefly, around 250 mg of frozen postmortem brain tissue was thawed in 5 ml lysis buffer and transferred to a douncer placed on ice. After 20–30 strokes, the homogenized tissue was transferred to a clear 50 ml ultracentrifuge tube and the volume was adjusted to 12 ml. 21 ml of sucrose buffer was added to the bottom of the clear ultracentrifuge tube, to create a concentration gradient with the homogenized tissue solution on top of the sucrose buffer. The tubes were placed in buckets in a SW32Ti swinging rotor (Beckton Dickinson). The samples were ultracentrifuged at 107163*g* for 2.5 h at 4 °C. The supernatant was removed and 500 μl of 1X PBS was added to the pellet and incubated for 20 min on ice. The nuclei were then resuspended and transferred into a microcentrifuge tube. The nuclei were counted using trypan blue dilution and then centrifuged at 500*g* for 5 min. The lysis buffer comprised 0.32 M sucrose, 5 mM CaCl_2_, 3 mM Mg(acetate)_2_, 0.1 mM EDTA, 10 mM Tris–HCl pH8, 1 mM DTT, 0.1%Triton X-100 in H_2_O. The sucrose buffer comprised 1.8 M sucrose, 3 mM Mg(acetate)_2_, 1 mM DTT, 10 mM Tris–HCl, pH8 in H_2_O.

### Immunostaining and sorting of the nuclei

The nuclei were resuspended at a concentration of 200,000 cells in 50 μl of 0.5% BSA in 1× PBS solution and stained for 45 min with Anti-NeuN Antibody Alexa Fluor 488(EMD Millipore) at a concentration of 1:400, and Anti-Maf antibody PE (BD biosciences) at a concentration of 1:50. The nuclei were then washed and strained using a 40 µm strainer. The sorting was done on an Aria II sorter using a 100 µm nozzle (data collected using FACSDIVA version 8.0.1 or earlier, BD Pharmingen). The nuclei were collected in 0.5% BSA in 1× PBS solution and centrifuged at 500*g* for 5 min. Flow cytometry analyses were performed using FlowJo vlO from BD Biosciences.

### DNA extraction, amplification and sequencing

DNA was extracted from the nuclei using the Qiagen QIAmp DNA micro kit. DNA concentration was measured using the Qubit fluorometer. PCR was performed to amplify the region surrounding the mutation of interest (around 300 bp) using the Phusion high-fidelity master mix (New England Biolabs). The amplified DNA was purified using the Qiagen QIAquick PCR purification kit according to the manufacturer’s recommendations. Libraries were generated from the pooled amplicons using the Celero DNA-seq library kit (NuGEN). Sequencing of the libraries was performed using MiSeq Nano v2 kits. Sequencing reads were aligned with BWA (http://bio-bwa.sourceforge.net), and variant calling and annotation were done with Varscan (http://varscan.sourceforge.net) and Annovar (https://annovar.openbioinformatics.org/en/latest/).

To confirm the reproducibility of the VAF measurements, DNA from unsorted brain nuclei or sorted Maf^+^ NeuN^−^ was used for library prep and sequencing in a second replicate. To rule out sequencing errors or other technical artifacts that could be artificially inflating the mutant allele fraction, we also performed amplicon sequencing using the same primers on a negative control brain sample from a donor known to not be a CHIP carrier. Primer sequences can be found in Supplementary Table 17. The VAFs of the unsorted brain and the sorted subsets were obtained from the same tissue prep for each sample, and the results can be found in Supplementary Table 14. For some samples, the same tissue prep was also used for snATAC-seq. However, in some cases, the tissue prep used for snATAC-seq was from a different piece of tissue from the same donor. In these cases, the VAFs were determined again using the same tissue prep as used for snATAC-seq, and the VAFs can be found in Supplementary Table 16.

### Single-nucleus ATAC-sequencing

#### Sample processing

After nuclei isolation as described above, samples were transposed, single cells were barcoded using 10X Genomics GEMs (Gel Bead in-EMulsions) and libraries were prepared for sequencing according to the commercially available 10X Chromium Next GEM Single Cell ATAC Library & Gel Bead Kit v1.1. Paired-end sequencing was performed on an Illumina HiSeq 2500. We performed single-nucleus ATAC-sequencing (snATAC-seq) on occipital cortex samples from all six donors with detected CHIP mutations in the brain, as well as the four donors without CHIP. We also performed snATAC-seq on putamen and cerebellum samples from donor ACT6.

#### Reference datasets

To aid in the interpretation of cell types from our snATAC-seq data, we also included two previously published datasets^24^. For the Corces et al. brain dataset, original fastq files of all ten snATAC samples (available under GEO accession no. GSE147672) were aligned to the hg19 reference genome and then processed as described above^55^. For the Satpathy et al. hematopoiesis dataset, we downloaded fragments files for the seven samples most relevant to our study, focusing on dendritic cells and monocytes^24^. Accession numbers and sample names of these are as follows:

GSM3722015_PBMC_Rep1_fragments.tsv.gz

GSM3722076_PBMC_Rep2_fragments.tsv.gz

GSM3722075_PBMC_Rep3_fragments.tsv.gz

GSM3722077_PBMC_Rep4_fragments.tsv.gz

GSM3722039_Dendritic_all_cells_fragments.tsv.gz

GSM3722026_Dendritic_Cells_fragments-Reformat.tsv.gz

GSM3722027_Monocytes_fragments.tsv.gz

#### Analysis pipeline

Fastq files were trimmed, deduplicated, filtered and aligned using the 10X cellranger-atac count pipeline, yielding a file of high-quality ATAC-seq fragments for all cells per sample. Reference genome hg19 was used for compatibility with the hematopoiesis reference dataset (described in ‘Reference datasets’)^25^. The fragments file for each sample, including the reference samples, was then loaded into ArchR for downstream analysis^55^.

Nuclei quality control and clustering were performed using the standard ArchR pipeline. Briefly, barcodes were called nuclei based on fragments per barcode and enrichment of fragments in transcription start sites (TSS) genome-wide. Doublets were removed in two steps. First, for each sample, doublets were predicted and removed based on similarity to computationally simulated doublets. Second, after initial clustering, we identified a cluster of putative doublets that was weakly enriched for markers of multiple cell types. This cluster was removed and the remaining nuclei were reprocessed and reclustered. The TileMatrix and GeneScoreMatrix were computed using default settings. For the GeneScoreMatrix, imputation was performed using the ArchR implementation of MAGIC to aid visualization of the sparse ATAC-seq signals in single nuclei. Dimensionality reduction and clustering were performed using the TileMatrix, which tiles the genome into 500 bp windows. Although Harmony batch correction is implemented and part of the standard workflow in ArchR, we did not use any batch correction to ensure that any biological differences between the samples would be preserved. After clustering, reproducible peaks were determined for each cluster individually to ensure that cell type-specific peaks were retained. Reproducible peaks for each cluster were merged into a set of disjoint, fixed width (500 bp) peaks, which were used to create the cell by peak matrix. ATAC-seq pseudo-bulk tracks for selected groups of nuclei were exported from ArchR using the ‘getGroupBW’ function. All tracks were identically normalized using ReadsInTSS, which corrects for variation in sequencing depth and also nuclei quality between different groups of nuclei. Specific regions in the genome were visualized using the Integrative Genomics Viewer (https://software.broadinstitute.org/software/igv/).

To evaluate the fraction of mutated MG (as shown in Fig. 5d and Supplementary Table 16), we first determined the number of MG and nonmicroglial hematopoietic cells in each brain sample. We assumed that all nonmicroglial hematopoietic cells were mutant, therefore we subtracted out the expected contribution of mutant alleles from these nonmicroglial cells to calculate an adjusted VAF for each sample where mutant alleles could only be contributed by MG. We then divided the adjusted percent mutant cells in each sample by the percent MG in each sample to calculate the percent mutant MG. An example calculation using the ACT3 sample is provided below.

vaf = 0.035 # VAF of the *DNMT3A* mutation from ACT3 unsorted brain

non_mg = 64 # Number of monocyte+DC+T-cell+B-cell in the sample

mg = 801 # Number of MG in the sample

total = 11,762 # Total number of nuclei identified in snATAC-seq

# We assume that only hematopoietic cells can carry the CHIP variant.

# We calculate the total number of mutant MG by

# multiplying the total*VAF*2 (gives total mutant cell burden) and

# subtracting all the nonmicroglial hematopoietic cells (assuming they

# are all mutant), which in this case is calculated to be 759.

mut_mg = round(total*vaf*2-non_mg)

# We calculate the proportion of mutant MG by dividing the

# number of mutant MG calculated above by the total number of

# MG. The value is 94.8%. Without accounting for the non

# microglial hematopoietic cells, the value would be vaf*total*2/mg,

# or 103%.

prop_mut_mg = mut_mg/mg

To model the expected distribution of the percentages of mutant MG based on this calculation, we simulated a binomial model for both the distribution of VAF obtained from amplicon sequencing and percentage of MG in unsorted brain nuclei. For this simulation, we assumed 200,000 haploid genomes (representing 100,000 starting nuclei, which is the minimum number used in our studies) and 8,000 nuclei assessed by snATAC-seq (representing approximately the median value in our samples). We varied the VAF based on the expected percent MG ranging from 10% to 90% and varied the percent MG in each sample from 1% to 3%. We then obtained histograms and 95% confidence intervals for each set of input parameters. These simulations indicated that the confidence intervals for the estimate of mutant MG are not overly broad based on expected input parameters. We then estimated bootstrapped confidence intervals for percent mutant MG for each sample using a similar simulation based on input parameters for VAF, percent MG, and a number of cells unique to each sample.

### Reporting summary

Further information on research design is available in the Nature Portfolio Reporting Summary linked to this article.

## Online content

Any methods, additional references, Nature Portfolio reporting summaries, source data, extended data, supplementary information, acknowledgements, peer review information; details of author contributions and competing interests; and statements of data and code availability are available at 10.1038/s41591-023-02397-2.

## Supplementary information

Supplementary Information Supplementary Appendix—TOPMed Consortium author list.

Reporting Summary

Supplementary Tables Supplementary Tables 1–17.

## Data Availability

Individual whole-genome sequencing data and individual-level harmonized phenotypes from TOPMed are available through dbGaP for investigators who follow dbGaP procedures for requesting controlled access data, as detailed at https://www.ncbi.nlm.nih.gov/projects/gap/cgi-bin/about.html#request-controlled. All whole exome sequencing data and phenotype data from ADSP are available on dbGaP for investigators who apply for access through https://www.niagads.org. dbGaP accession numbers: https://www.ncbi.nlm.nih.gov/projects/gap/cgi-bin/study.cgi?study_id=phs000974.v1.p1 https://www.ncbi.nlm.nih.gov/projects/gap/cgi-bin/study.cgi?study_id=phs001368.v2.p2 https://www.ncbi.nlm.nih.gov/projects/gap/cgi-bin/study.cgi?study_id=phs000572.v8.p4 Single-nucleus ATAC-seq data from human brain samples are available in Gene Expression Omnibus under accession GSE192838.

## Code availability

Code and additional metadata tables for the snATAC-seq data are available at https://github.com/juliabelk/CHIP_and_AD (10.5281/zenodo.7809346).
