# Supplementary material for: Clonal hematopoiesis is associated with protection from Alzheimer’s disease
Source: Nat Med. 2023 Jun 15;29(7):1662–70. doi: 10.1038/s41591-023-02397-2 (PMC10353941; doi:10.1038/s41591-023-02397-2)
Supplement: Supplementary file 1 — Supplementary Appendix—TOPMed Consortium author list. [file 41591_2023_2397_MOESM1_ESM.pdf]

nature medicine

Article

https://doi.org/10.1038/s41591-023-02397-2

Clonal hematopoiesis is associated with protection from Alzheimer's disease

In the format provided by the authors and unedited

Supplementary Appendix
TOPMed consortium full author list

Name	Institution(s)	Primary Department	Institution City	Institution State	Zip Code	Country
Abe, Namiko	New York Genome Center		New York	New York	10013	US
Abecasis, Gonçalo	University of Michigan		Ann Arbor	Michigan	48109	US
Aguet, Francois	Broad Institute		Cambridge	Massachusetts	02142	US
Albert, Christine	Cedars Sinai		Boston	Massachusetts	02114	US
Almasy, Laura	Children's Hospital of Philadelphia, University of Pennsylvania		Philadelphia	Pennsylvania	19104	US
Alonso, Alvaro	Emory University		Atlanta	Georgia	30322	US
Ament, Seth	University of Maryland		Baltimore	Maryland	21201	US
Anderson, Peter	University of Washington		Seattle	Washington	98195	US
Anugu, Pramod	University of Mississippi		Jackson	Mississippi	38677	US
Applebaum-Bowden, Deborah	National Institutes of Health		Bethesda	Maryland	20892	US
Ardlie, Kristin	Broad Institute		Cambridge	Massachusetts	02142	US
Arking, Dan	Johns Hopkins University		Baltimore	Maryland	21218	US
Arnett, Donna K	University of South Carolina					US
Ashley-Koch, Allison	Duke University		Durham	North Carolina	27708	US
Aslibekyan, Stella	University of Alabama		Birmingham	Alabama	35487	US

Name	Institution(s)	Primary Department	Institution City	Institution State	Zip Code	Country
Assimes, Tim	Stanford University		Stanford	California	94305	US
Auer, Paul	Medical College of Wisconsin		Milwaukee	Wisconsin	53211	US
Avramopoulos, Dimitrios	Johns Hopkins University		Baltimore	Maryland	21218	US
Ayas, Najib	Providence Health Care	Medicine	Vancouver			CA
Balasubramanian, Adithya	Baylor College of Medicine Human Genome Sequencing Center		Houston	Texas	77030	US
Barnard, John	Cleveland Clinic		Cleveland	Ohio	44195	US
Barnes, Kathleen	Tempus, University of Colorado Anschutz Medical Campus		Aurora	Colorado	80045	US
Barr, R. Graham	Columbia University		New York	New York	10032	US
Barron-Casella, Emily	Johns Hopkins University		Baltimore	Maryland	21218	US
Barwick, Lucas	The Emmes Corporation	LTRC	Rockville	Maryland	20850	US
Beaty, Terri	Johns Hopkins University		Baltimore	Maryland	21218	US
Beck, Gerald	Cleveland Clinic	Quantitative Health Sciences	Cleveland	Ohio	44195	US
Becker, Diane	Johns Hopkins University	Medicine	Baltimore	Maryland	21218	US

Name	Institution(s)	Primary Department	Institution City	Institution State	Zip Code	Country
Becker, Lewis	Johns Hopkins University		Baltimore	Maryland	21218	US
Beer, Rebecca	National Heart, Lung, and Blood Institute, National Institutes of Health		Bethesda	Maryland	20892	US
Beitelshees, Amber	University of Maryland		Baltimore	Maryland	21201	US
Benjamin, Emelia	Boston University, Massachusetts General Hospital	Boston University School of Medicine	Boston	Massachusetts	02114	US
Benos, Takis	University of Florida		Pittsburgh	Pennsylvania	15260	US
Bezerra, Marcos	Fundação de Hematologia e Hemoterapia de Pernambuco - Hemope		Recife		52011-000	BR
Bielak, Larry	University of Michigan		Ann Arbor	Michigan	48109	US
Bis, Joshua	University of Washington	Cardiovascular Health Research Unit, Department of Medicine	Seattle	Washington	98195	US
Blackwell, Thomas	University of Michigan		Ann Arbor	Michigan	48109	US
Blangero, John	University of Texas Rio Grande Valley School of Medicine	Human Genetics	Brownsville	Texas	78520	US
Blue, Nathan	University of Utah	Obstetrics and Gynecology	Salt Lake City	Utah	84132	US

Name	Institution(s)	Primary Department	Institution City	Institution State	Zip Code	Country
Boerwinkle, Eric	University of Texas Health at Houston		Houston	Texas	77225	US
Bowden, Donald W.	Wake Forest Baptist Health	Department of Biochemistry	Winston-Salem	North Carolina	27157	US
Bowler, Russell	National Jewish Health	National Jewish Health	Denver	Colorado	80206	US
Brody, Jennifer	University of Washington		Seattle	Washington	98195	US
Broeckel, Ulrich	Medical College of Wisconsin	Pediatrics	Milwaukee	Wisconsin	53226	US
Broome, Jai	University of Washington		Seattle	Washington	98195	US
Brown, Deborah	University of Texas Health at Houston	Pediatrics	Houston	Texas	77030	US
Bunting, Karen	New York Genome Center		New York	New York	10013	US
Burchard, Esteban	University of California, San Francisco		San Francisco	California	94143	US
Bustamante, Carlos	Stanford University	Biomedical Data Science	Stanford	California	94305	US
Buth, Erin	University of Washington	Biostatistics	Seattle	Washington	98195	US
Cade, Brian	Brigham & Women's Hospital	Brigham and Women's Hospital	Boston	Massachusetts	02115	US
Cardwell, Jonathan	University of Colorado at Denver		Denver	Colorado	80204	US
Carey, Vincent	Brigham & Women's Hospital		Boston	Massachusetts	02115	US
Carrier, Julie	University of Montreal					US
Carson, April P.	University of Mississippi	Medicine	Jackson	Mississippi	39213	US

Name	Institution(s)	Primary Department	Institution City	Institution State	Zip Code	Country
Carty, Cara	Washington State University		Pullman	Washington	99164	US
Casaburi, Richard	University of California, Los Angeles		Los Angeles	California	90095	US
Casas Romero, Juan P	Brigham & Women's Hospital					US
Casella, James	Johns Hopkins University		Baltimore	Maryland	21218	US
Castaldi, Peter	Brigham & Women's Hospital	Medicine	Boston	Massachusetts	02115	US
Chaffin, Mark	Broad Institute		Cambridge	Massachusetts	02142	US
Chang, Christy	University of Maryland		Baltimore	Maryland	21201	US
Chang, Yi-Cheng	National Taiwan University		Taipei		10617	TW
Chasman, Daniel	Brigham & Women's Hospital	Division of Preventive Medicine	Boston	Massachusetts	02215	US
Chavan, Sameer	University of Colorado at Denver		Denver	Colorado	80204	US
Chen, Bo-Juen	New York Genome Center		New York	New York	10013	US
Chen, Wei-Min	University of Virginia		Charlottesville	Virginia	22903	US
Chen, Yii-Der Ida	Lundquist Institute		Torrance	California	90502	US
Cho, Michael	Brigham & Women's Hospital		Boston	Massachusetts	02115	US
Choi, Seung Hoan	Broad Institute		Cambridge	Massachusetts	02142	US

Name	Institution(s)	Primary Department	Institution City	Institution State	Zip Code	Country
Chuang, Lee-Ming	National Taiwan University	National Taiwan University Hospital	Taipei		10617	TW
Chung, Mina	Cleveland Clinic	Cleveland Clinic	Cleveland	Ohio	44195	US
Chung, Ren-Hua	National Health Research Institute Taiwan		Miaoli County		350	TW
Clish, Clary	Broad Institute	Metabolomics Platform	Cambridge	Massachusetts	02142	US
Comhair, Suzy	Cleveland Clinic	Immunity and Immunology	Cleveland	Ohio	44195	US
Conomos, Matthew	University of Washington	Biostatistics	Seattle	Washington	98195	US
Cornell, Elaine	University of Vermont		Burlington	Vermont	05405	US
Correa, Adolfo	University of Mississippi	Population Health Science	Jackson	Mississippi	39216	US
Crandall, Carolyn	University of California, Los Angeles		Los Angeles	California	90095	US
Crapo, James	National Jewish Health		Denver	Colorado	80206	US
Cupples, L. Adrienne	Boston University	Biostatistics	Boston	Massachusetts	02115	US
Curran, Joanne	University of Texas Rio Grande Valley School of Medicine		Brownsville	Texas	78520	US
Curtis, Jeffrey	University of Michigan	Internal Medicine	Ann Arbor	Michigan	48109	US
Custer, Brian	Vitalant Research Institute		San Francisco	California	94118	US
Damcott, Coleen	University of Maryland		Baltimore	Maryland	21201	US

Name	Institution(s)	Primary Department	Institution City	Institution State	Zip Code	Country
Darbar, Dawood	University of Illinois at Chicago		Chicago	Illinois	60607	US
David, Sean	University of Chicago		Chicago	Illinois	60637	US
Davis, Colleen	University of Washington		Seattle	Washington	98195	US
Daya, Michelle	University of Colorado at Denver		Denver	Colorado	80204	US
de Andrade, Mariza	Mayo Clinic	Health Quantitative Sciences Research	Rochester	Minnesota	55905	US
de las Fuentes, Lisa	Washington University in St Louis	Department of Medicine, Cardiovascular Division	St. Louis	Missouri	63110	US
de Vries, Paul	University of Texas Health at Houston	Human Genetics Center, Department of Epidemiology, Human Genetics, and Environmental Sciences	Houston	Texas	77030	US
DeBaun, Michael	Vanderbilt University		Nashville	Tennessee	37235	US
Deka, Ranjan	University of Cincinnati		Cincinnati	Ohio	45220	US
DeMeo, Dawn	Brigham & Women's Hospital		Boston	Massachusetts	02115	US
Devine, Scott	University of Maryland		Baltimore	Maryland	21201	US
Dinh, Huyen	Baylor College of Medicine Human Genome Sequencing Center		Houston	Texas	77030	US
Doddapaneni, Harsha	Baylor College of Medicine		Houston	Texas	77030	

Name	Institution(s)	Primary Department	Institution City	Institution State	Zip Code	Country
Duan, Qing	Human Genome Sequencing Center University of North Carolina		Chapel Hill	North Carolina	27599	US
Dugan-Perez, Shannon	Baylor College of Medicine Human Genome Sequencing Center	BCM	Houston	Texas	77030	US
Duggirala, Ravi	University of Texas Rio Grande Valley School of Medicine		Edinburg	Texas	78539	US
Durda, Jon Peter	University of Vermont	Pathology and Laboratory Medicine	Burlington	Vermont	05405	US
Dutcher, Susan K.	Washington University in St Louis	Genetics	St Louis	Missouri	63110	US
Eaton, Charles	Brown University		Providence	Rhode Island	02912	US
Ekunwe, Lynette	University of Mississippi		Jackson	Mississippi	38677	US
El Boueiz, Adel	Harvard University	Channing Division of Network Medicine	Cambridge	Massachusetts	02138	US
Ellinor, Patrick	Massachusetts General Hospital		Boston	Massachusetts	02114	US
Emery, Leslie	University of Washington		Seattle	Washington	98195	US
Erzurum, Serpil	Cleveland Clinic	Lerner Research Institute	Cleveland	Ohio	44195	US
Farber, Charles	University of Virginia		Charlottesville	Virginia	22903	US
Farek, Jesse	Baylor College of		Houston	Texas	77030	US

Name	Institution(s)	Primary Department	Institution City	Institution State	Zip Code	Country
	Medicine Human Genome Sequencing Center					
Fingerlin, Tasha	National Jewish Health	Center for Genes, Environment and Health	Denver	Colorado	80206	US
Flickinger, Matthew	University of Michigan		Ann Arbor	Michigan	48109	US
Fornage, Myriam	University of Texas Health at Houston	Institute of Molecular Medicine McGovern Medical School	Houston	Texas	77225	US
Franceschini, Nora	University of North Carolina	Epidemiology	Chapel Hill	North Carolina	27599	US
Frazar, Chris	University of Washington		Seattle	Washington	98195	US
Fu, Mao	University of Maryland		Baltimore	Maryland	21201	US
Fullerton, Stephanie M.	University of Washington		Seattle	Washington	98195	US
Fulton, Lucinda	Washington University in St Louis		St Louis	Missouri	63130	US
Gabriel, Stacey	Broad Institute		Cambridge	Massachusetts	02142	US
Gan, Weiniu	National Heart, Lung, and Blood Institute, National Institutes of Health		Bethesda	Maryland	20892	US
Gao, Shanshan	University of Colorado at Denver		Denver	Colorado	80204	US
Gao, Yan	University of Mississippi		Jackson	Mississippi	38677	US
Gass, Margery	Fred Hutchinson		Seattle	Washington	98109	US

Name	Institution(s)	Primary Department	Institution City	Institution State	Zip Code	Country
	Cancer Research Center					
Geiger, Heather	New York Genome Center		New York City	New York	10013	US
Gelb, Bruce	Icahn School of Medicine at Mount Sinai		New York	New York	10029	US
Geraci, Mark	University of Pittsburgh		Pittsburgh	Pennsylvania		US
Germer, Soren	New York Genome Center		New York	New York	10013	US
Gerszten, Robert	Beth Israel Deaconess Medical Center		Boston	Massachusetts	02215	US
Ghosh, Auyon	Brigham & Women's Hospital		Boston	Massachusetts	02115	US
Gibbs, Richard	Baylor College of Medicine Human Genome Sequencing Center		Houston	Texas	77030	US
Gignoux, Chris	Stanford University		Stanford	California	94305	US
Gladwin, Mark	University of Pittsburgh		Pittsburgh	Pennsylvania	15260	US
Glahn, David	Boston Children's Hospital, Harvard Medical School	Department of Psychiatry	Boston	Massachusetts	02115	US
Gogarten, Stephanie	University of Washington		Seattle	Washington	98195	US

Name	Institution(s)	Primary Department	Institution City	Institution State	Zip Code	Country
Gong, Da-Wei	University of Maryland		Baltimore	Maryland	21201	US
Goring, Harald	University of Texas Rio Grande Valley School of Medicine		San Antonio	Texas	78229	US
Graw, Sharon	University of Colorado Anschutz Medical Campus		Aurora	Colorado	80045	US
Gray, Kathryn J.	Mass General Brigham	Obstetrics and Gynecology	Boston	Massachusetts	02115	US
Grine, Daniel	University of Colorado at Denver		Denver	Colorado	80204	US
Gross, Colin	University of Michigan		Ann Arbor	Michigan	48109	US
Gu, C. Charles	Washington University in St Louis		St Louis	Missouri	63130	US
Guan, Yue	University of Maryland		Baltimore	Maryland	21201	US
Guo, Xiuqing	Lundquist Institute		Torrance	California	90502	US
Gupta, Namrata	Broad Institute	Broad Institute	Cambridge	Massachusetts	02142	US
Haessler, Jeff	Fred Hutchinson Cancer Research Center		Seattle	Washington	98109	US
Hall, Michael	University of Mississippi	Cardiology	Jackson	Mississippi	39216	US
Han, Yi	Baylor College of Medicine Human Genome		Houston	Texas	77030	US

Name	Institution(s)	Primary Department	Institution City	Institution State	Zip Code	Country
	Sequencing Center					
Hanly, Patrick	University of Calgary	Medicine	Calgary			CA
Harris, Daniel	University of Maryland	Genetics	Philadelphia	Pennsylvania	19104	US
Hawley, Nicola L.	Yale University	Department of Chronic Disease Epidemiology	New Haven	Connecticut	06520	US
He, Jiang	Tulane University		New Orleans	Louisiana	70118	US
Heavner, Ben	University of Washington	Biostatistics	Seattle	Washington	98195	US
Heckbert, Susan	University of Washington	Epidemiology	Seattle	Washington	98195-9458	US
Hernandez, Ryan	University of California, San Francisco		San Francisco	California	94143	US
Herrington, David	Wake Forest Baptist Health		Winston-Salem	North Carolina	27157	US
Hersh, Craig	Brigham & Women's Hospital	Channing Division of Network Medicine	Boston	Massachusetts	02115	US
Hidalgo, Bertha	University of Alabama		Birmingham	Alabama	35487	US
Hixson, James	University of Texas Health at Houston		Houston	Texas	77225	US
Hobbs, Brian	Brigham & Women's Hospital		Boston	Massachusetts	02115	US
Hokanson, John	University of Colorado at Denver		Denver	Colorado	80204	US
Hong, Elliott	University of Maryland		Baltimore	Maryland	21201	US
Hoth, Karin	University of Iowa		Iowa City	Iowa	52242	US

Name	Institution(s)	Primary Department	Institution City	Institution State	Zip Code	Country
Hsiung, Chao (Agnes)	National Health Research Institute Institute Taiwan	Institute of Population Health Sciences, NHRI	Miaoli County		350	TW
Hu, Jianhong	Baylor College of Medicine Human Genome Sequencing Center		Houston	Texas	77030	US
Hung, Yi-Jen	Tri-Service General Hospital National Defense Medical Center					TW
Huston, Haley	Blood Works Northwest		Seattle	Washington	98104	US
Hwu, Chii Min	Taichung Veterans General Hospital Taiwan		Taichung City		407	TW
Irvin, Marguerite Ryan	University of Alabama		Birmingham	Alabama	35487	US
Jackson, Rebecca	Oklahoma State University Medical Center	Internal Medicine, DIvision of Endocrinology, Diabetes and Metabolism	Columbus	Ohio	43210	US
Jain, Deepti	University of Washington		Seattle	Washington	98195	US
Jaquish, Cashell	National Heart, Lung, and Blood Institute, National Institutes of Health	NHLBI	Bethesda	Maryland	20892	US

Name	Institution(s)	Primary Department	Institution City	Institution State	Zip Code	Country
Johnsen, Jill	University of Washington	Medicine	Seattle	Washington	98109	US
Johnson, Andrew	National Heart, Lung, and Blood Institute, National Institutes of Health		Bethesda	Maryland	20892	US
Johnson, Craig	University of Washington		Seattle	Washington	98195	US
Johnston, Rich	Emory University		Atlanta	Georgia	30322	US
Jones, Kimberly	Johns Hopkins University		Baltimore	Maryland	21218	US
Kang, Hyun Min	University of Michigan	Biostatistics	Ann Arbor	Michigan	48109	US
Kaplan, Robert	Albert Einstein College of Medicine		New York	New York	10461	US
Kardia, Sharon	University of Michigan		Ann Arbor	Michigan	48109	US
Kelly, Shannon	University of California, San Francisco		San Francisco	California	94118	US
Kenny, Eimear	Icahn School of Medicine at Mount Sinai		New York	New York	10029	US
Kessler, Michael	University of Maryland		Baltimore	Maryland	21201	US
Khan, Alyna	University of Washington		Seattle	Washington	98195	US
Khan, Ziad	Baylor College of Medicine Human Genome		Houston	Texas	77030	US

Name	Institution(s)	Primary Department	Institution City	Institution State	Zip Code	Country
	Sequencing Center					
Kim, Wonji	Harvard University		Cambridge	Massachusetts	02138	US
Kimoff, John	McGill University		Montréal		QC H3A 0G4	CA
Kinney, Greg	University of Colorado at Denver	Epidemiology	Aurora	Colorado	80045	US
Konkle, Barbara	Blood Works Northwest	Medicine	Seattle	Washington	98104	US
Kooperberg, Charles	Fred Hutchinson Cancer Research Center		Seattle	Washington	98109	US
Kramer, Holly	Loyola University	Public Health Sciences	Maywood	Illinois	60153	US
Lange, Christoph	Harvard School of Public Health	Biostats	Boston	Massachusetts	02115	US
Lange, Ethan	University of Colorado at Denver		Denver	Colorado	80204	US
Lange, Leslie	University of Colorado at Denver	Medicine	Aurora	Colorado	80048	US
Laurie, Cathy	University of Washington		Seattle	Washington	98195	US
Laurie, Cecelia	University of Washington		Seattle	Washington	98195	US
LeBoff, Meryl	Brigham & Women's Hospital		Boston	Massachusetts	02115	US
Lee, Jiwon	Brigham & Women's Hospital		Boston	Massachusetts	02115	US
Lee, Sandra	Baylor College of Medicine Human		Houston	Texas	77030	US

Name	Institution(s)	Primary Department	Institution City	Institution State	Zip Code	Country
	Genome Sequencing Center					
Lee, Wen-Jane	Taichung Veterans General Hospital Taiwan		Taichung City		407	TW
LeFaive, Jonathon	University of Michigan		Ann Arbor	Michigan	48109	US
Levine, David	University of Washington		Seattle	Washington	98195	US
Levy, Dan	National Heart, Lung, and Blood Institute, National Institutes of Health		Bethesda	Maryland	20892	US
Lewis, Joshua	University of Maryland		Baltimore	Maryland	21201	US
Li, Xiaohui	Lundquist Institute		Torrance	California	90502	US
Li, Yun	University of North Carolina		Chapel Hill	North Carolina	27599	US
Lin, Henry	Lundquist Institute		Torrance	California	90502	US
Lin, Honghuang	Boston University	University of Massachusetts Chan Medical School	Worcester	Massachusetts	01655	US
Lin, Xihong	Harvard School of Public Health	Dept of Biostatistics	Boston	Massachusetts	02115	US
Liu, Simin	Brown University	Epidemiology and Medicine	Providence	Rhode Island	02912	US
Liu, Yongmei	Duke University	Cardiology	Durham	North Carolina	27708	US
Liu, Yu	Stanford University	Cardiovascular Institute	Stanford	California	94305	US

Name	Institution(s)	Primary Department	Institution City	Institution State	Zip Code	Country
Loos, Ruth J.F.	Icahn School of Medicine at Mount Sinai	The Charles Bronfman Institute for Personalized Medicine	New York	New York	10029	US
Lubitz, Steven	Massachusetts General Hospital		Boston	Massachusetts	02114	US
Lunetta, Kathryn	Boston University		Boston	Massachusetts	02215	US
Luo, James	National Heart, Lung, and Blood Institute, National Institutes of Health		Bethesda	Maryland	20892	US
Magalang, Ulysses	The Ohio State University	Division of Pulmonary, Critical Care and Sleep Medicine	Columbus	Ohio	43210	US
Mahaney, Michael	University of Texas Rio Grande Valley School of Medicine		Brownsville	Texas	78520	US
Make, Barry	Johns Hopkins University		Baltimore	Maryland	21218	US
Manichaikul, Ani	University of Virginia		Charlottesville	Virginia	22903	US
Manning, Alisa	Broad Institute, Harvard University, Massachusetts General Hospital					
Manson, JoAnn	Brigham & Women's Hospital		Boston	Massachusetts	02115	US

Name	Institution(s)	Primary Department	Institution City	Institution State	Zip Code	Country
Martin, Lisa	George Washington University	cardiology	Washington	District of Columbia	20037	US
Marton, Melissa	New York Genome Center		New York City	New York	10013	US
Mathai, Susan	University of Colorado at Denver		Denver	Colorado	80204	US
Mathias, Rasika	Johns Hopkins University		Baltimore	Maryland	21218	US
May, Susanne	University of Washington	Biostatistics	Seattle	Washington	98195	US
McArdle, Patrick	University of Maryland		Baltimore	Maryland	21201	US
McDonald, Merry-Lynn	University of Alabama	University of Alabama at Birmingham	Birmingham	Alabama	35487	US
McFarland, Sean	Harvard University		Cambridge	Massachusetts	02138	US
McGarvey, Stephen	Brown University	Epidemiology	Providence	Rhode Island	02912	US
McGoldrick, Daniel	University of Washington	Genome Sciences	Seattle	Washington	98195	US
McHugh, Caitlin	University of Washington	Biostatistics	Seattle	Washington	98195	US
McNeil, Becky	RTI International					US
Mei, Hao	University of Mississippi		Jackson	Mississippi	38677	US
Meigs, James	Massachusetts General Hospital	Medicine	Boston	Massachusetts	02114	US
Menon, Vipin	Baylor College of Medicine Human Genome Sequencing Center		Houston	Texas	77030	US

Name	Institution(s)	Primary Department	Institution City	Institution State	Zip Code	Country
Mestroni, Luisa	University of Colorado Anschutz Medical Campus		Aurora	Colorado	80045	US
Metcalf, Ginger	Baylor College of Medicine Human Genome Sequencing Center		Houston	Texas	77030	US
Meyers, Deborah A	University of Arizona		Tucson	Arizona	85721	US
Mignot, Emmanuel	Stanford University	Center For Sleep Sciences and Medicine	Palo Alto	California	94304	US
Mikulla, Julie	National Heart, Lung, and Blood Institute, National Institutes of Health		Bethesda	Maryland	20892	US
Min, Nancy	University of Mississippi		Jackson	Mississippi	38677	US
Minear, Mollie	National Institute of Child Health and Human Development, National Institutes of Health		Bethesda	Maryland	20892	US
Minster, Ryan L	University of Pittsburgh		Pittsburgh	Pennsylvania	15260	US
Mitchell, Braxton D.	University of Maryland		Baltimore	Maryland	21201	US
Moll, Matt	Brigham & Women's Hospital	Medicine	Boston	Massachusetts	02115	US

Name	Institution(s)	Primary Department	Institution City	Institution State	Zip Code	Country
Momin, Zeineen	Baylor College of Medicine Human Genome Sequencing Center		Houston	Texas	77030	US
Montasser, May E.	University of Maryland		Baltimore	Maryland	21201	US
Montgomery, Courtney	Oklahoma Medical Research Foundation	Genes and Human Disease	Oklahoma City	Oklahoma	73104	US
Muzny, Donna	Baylor College of Medicine Human Genome Sequencing Center		Houston	Texas	77030	US
Mychaleckyj, Josyf C	University of Virginia		Charlottesville	Virginia	22903	US
Nadkarni, Girish	Icahn School of Medicine at Mount Sinai		New York	New York	10029	US
Naik, Rakhi	Johns Hopkins University		Baltimore	Maryland	21218	US
Naseri, Take	Ministry of Health, Government of Samoa		Apia			WS
Natarajan, Pradeep	Broad Institute		Cambridge	Massachusetts	02142	US
Nekhai, Sergei	Howard University		Washington	District of Columbia	20059	US
Nelson, Sarah C.	University of Washington	Biostatistics	Seattle	Washington	98195	US
Neltner, Bonnie	University of Colorado at Denver		Denver	Colorado	80204	US

Name	Institution(s)	Primary Department	Institution City	Institution State	Zip Code	Country
Nessner, Caitlin	Baylor College of Medicine Human Genome Sequencing Center		Houston	Texas	77030	US
Nickerson, Deborah	University of Washington	Department of Genome Sciences	Seattle	Washington	98195	US
Nkechinyere, Osuji	Baylor College of Medicine Human Genome Sequencing Center		Houston	Texas	77030	US
North, Kari	University of North Carolina		Chapel Hill	North Carolina	27599	US
O'Connell, Jeff	University of Maryland		Balitmore	Maryland	21201	US
O'Connor, Tim	University of Maryland		Baltimore	Maryland	21201	US
Ochs-Balcom, Heather	University at Buffalo		Buffalo	New York	14260	US
Okwuonu, Geoffrey	Baylor College of Medicine Human Genome Sequencing Center		Houston	Texas	77030	US
Pack, Allan	University of Pennsylvania	Division of Sleep Medicine/Department of Medicine	Philadelphia	Pennsylvania	19104-3403	US
Paik, David T.	Stanford University	Stanford Cardiovascular Institute	Stanford	California	94305	US
Palmer, Nicholette	Wake Forest Baptist Health	Biochemistry	Winston-Salem	North Carolina	27157	US
Pankow, James	University of Minnesota		Minneapolis	Minnesota	55455	US

Name	Institution(s)	Primary Department	Institution City	Institution State	Zip Code	Country
Papanicolaou, George	National Heart, Lung, and Blood Institute, National Institutes of Health		Bethesda	Maryland	20892	US
Parker, Cora	RTI International	Biostatistics and Epidemiology Division	Research Triangle Park	North Carolina	27709-2194	US
Peloso, Gina	Boston University	Department of Biostatistics	Boston	Massachusetts	02118	US
Peralta, Juan Manuel	University of Texas Rio Grande Valley School of Medicine		Edinburg	Texas	78539	US
Perez, Marco	Stanford University		Stanford	California	94305	US
Perry, James	University of Maryland		Baltimore	Maryland	21201	US
Peters, Ulrike	Fred Hutchinson Cancer Research Center	Fred Hutch and UW	Seattle	Washington	98109	US
Peyser, Patricia	University of Michigan		Ann Arbor	Michigan	48109	US
Phillips, Lawrence S	Emory University		Atlanta	Georgia	30322	US
Pleiness, Jacob	University of Michigan		Ann Arbor	Michigan	48109	US
Pollin, Toni	University of Maryland		Baltimore	Maryland	21201	US
Post, Wendy	Johns Hopkins University	Cardiology/Medicine	Baltimore	Maryland	21218	US
Powers Becker, Julia	University of Colorado at Denver	Medicine	Denver	Colorado	80204	US

Name	Institution(s)	Primary Department	Institution City	Institution State	Zip Code	Country
Preethi Boorgula, Meher	University of Colorado at Denver		Denver	Colorado	80204	US
Preuss, Michael	Icahn School of Medicine at Mount Sinai		New York	New York	10029	US
Psaty, Bruce	University of Washington		Seattle	Washington	98195	US
Qasba, Pankaj	National Heart, Lung, and Blood Institute, National Institutes of Health		Bethesda	Maryland	20892	US
Qiao, Dandi	Brigham & Women's Hospital		Boston	Massachusetts	02115	US
Qin, Zhaohui	Emory University		Atlanta	Georgia	30322	US
Rafaels, Nicholas	University of Colorado at Denver	CCPM	Denver	Colorado	80045	US
Raffield, Laura	University of North Carolina	Genetics	Chapel Hill	North Carolina	27599	US
Rajendran, Mahitha	Baylor College of Medicine Human Genome Sequencing Center		Houston	Texas	77030	US
Ramachandran, Vasan S.	Boston University		Boston	Massachusetts	02215	US
Rao, D.C.	Washington University in St Louis		St Louis	Missouri	63130	US
Rasmussen-Torvik, Laura	Northwestern University		Chicago	Illinois	60208	US

Name	Institution(s)	Primary Department	Institution City	Institution State	Zip Code	Country
Ratan, Aakrosh	University of Virginia		Charlottesville	Virginia	22903	US
Redline, Susan	Brigham & Women's Hospital	Medicine	Boston	Massachusetts	02115	US
Reed, Robert	University of Maryland		Baltimore	Maryland	21201	US
Reeves, Catherine	New York Genome Center	New York Genome Center	New York City	New York	10013	US
Regan, Elizabeth	National Jewish Health		Denver	Colorado	80206	US
Reiner, Alex	Fred Hutchinson Cancer Research Center, University of Washington		Seattle	Washington	98109	US
Reupena, Muagututi‘a Sefuiva	Lutia I Puava Ae Mapu I Fagalele		Apia			WS
Rice, Ken	University of Washington		Seattle	Washington	98195	US
Rich, Stephen	University of Virginia		Charlottesville	Virginia	22903	US
Robillard, Rebecca	University of Ottawa	Sleep Research Unit, University of Ottawa Institute for Mental Health Research	Ottawa		ON K1Z 7K4	CA
Robine, Nicolas	New York Genome Center		New York City	New York	10013	US
Roden, Dan	Vanderbilt University	Medicine, Pharmacology, Biomedicla Informatics	Nashville	Tennessee	37235	US
Roselli, Carolina	Broad Institute		Cambridge	Massachusetts	02142	US
Rotter, Jerome	Lundquist Institute	Pediatrics	Torrance	California	90502	US

Name	Institution(s)	Primary Department	Institution City	Institution State	Zip Code	Country
Ruczinski, Ingo	Johns Hopkins University		Baltimore	Maryland	21218	US
Runnels, Alexi	New York Genome Center		New York City	New York	10013	US
Russell, Pamela	University of Colorado at Denver		Denver	Colorado	80204	US
Ruuska, Sarah	University of Washington		Seattle	Washington	98104	US
Ryan, Kathleen	University of Maryland		Baltimore	Maryland	21201	US
Sabino, Ester Cerdeira	Universidade de Sao Paulo	Faculdade de Medicina	Sao Paulo		01310000	BR
Saleheen, Danish	Columbia University		New York	New York	10027	US
Salimi, Shabnam	University of Maryland	Pathology	Seattle	Washington	98195	US
Salvi, Sejal	Baylor College of Medicine Human Genome Sequencing Center		Houston	Texas	77030	US
Salzberg, Steven	Johns Hopkins University		Baltimore	Maryland	21218	US
Sandow, Kevin	Lundquist Institute	TGPS	Torrance	California	90502	US
Sankaran, Vijay G.	Harvard University	Division of Hematology/Oncology	Boston	Massachusetts	02115	US
Santibanez, Jireh	Baylor College of Medicine Human Genome Sequencing Center		Houston	Texas	77030	US

Name	Institution(s)	Primary Department	Institution City	Institution State	Zip Code	Country
Schwander, Karen	Washington University in St Louis		St Louis	Missouri	63130	US
Schwartz, David	University of Colorado at Denver		Denver	Colorado	80204	US
Sciurba, Frank	University of Pittsburgh		Pittsburgh	Pennsylvania	15260	US
Seidman, Christine	Harvard Medical School	Genetics	Boston	Massachusetts	02115	US
Seidman, Jonathan	Harvard Medical School		Boston	Massachusetts	02115	US
Sériès, Frédéric	Université Laval		Quebec City		G1V 0A6	CA
Sheehan, Vivien	Emory University	Pediatrics	Atlanta	Georgia	30307	US
Sherman, Stephanie L.	Emory University	Human Genetics	Atlanta	Georgia	30322	US
Shetty, Amol	University of Maryland		Baltimore	Maryland	21201	US
Shetty, Aniket	University of Colorado at Denver		Denver	Colorado	80204	US
Sheu, Wayne Hui-Heng	Taichung Veterans General Hospital Taiwan		Taichung City		407	TW
Shoemaker, M. Benjamin	Vanderbilt University	Medicine/Cardiology	Nashville	Tennessee	37235	US
Silver, Brian	UMass Memorial Medical Center		Worcester	Massachusetts	01655	US
Silverman, Edwin	Brigham & Women's Hospital		Boston	Massachusetts	02115	US
Skomro, Robert	University of Saskatchewan		Saskatoon		SK S7N 5C9	CA

Name	Institution(s)	Primary Department	Institution City	Institution State	Zip Code	Country
Smith, Albert Vernon	University of Michigan					
Smith, Jennifer	University of Michigan		Ann Arbor	Michigan	48109	US
Smith, Josh	University of Washington		Seattle	Washington	98195	US
Smith, Nicholas	University of Washington	Epidemiology	Seattle	Washington	98195	US
Smith, Tanja	New York Genome Center		New York	New York	10013	US
Smoller, Sylvia	Albert Einstein College of Medicine		New York	New York	10461	US
Snively, Beverly	Wake Forest Baptist Health	Biostatistical Sciences	Winston-Salem	North Carolina	27157	US
Snyder, Michael	Stanford University	Genetics	Stanford	California	94305	US
Sofer, Tamar	Beth Israel Deaconess Medical Center		Boston	Massachusetts	02215	US
Sotoodehnia, Nona	University of Washington		Seattle	Washington	98195	US
Stilp, Adrienne M.	University of Washington		Seattle	Washington	98195	US
Storm, Garrett	University of Colorado at Denver	Genomic Cardiology	Aurora	Colorado	80045	US
Streeten, Elizabeth	University of Maryland		Baltimore	Maryland	21201	US
Su, Jessica Lasky	Brigham & Women's Hospital	Channing Department of Medicine	Boston	Massachusetts	02115	US
Sung, Yun Ju	Washington University in St Louis		St Louis	Missouri	63130	US
Sylvia, Jody	Brigham & Women's Hospital		Boston	Massachusetts	02115	US

Name	Institution(s)	Primary Department	Institution City	Institution State	Zip Code	Country
Szpiro, Adam	University of Washington		Seattle	Washington	98195	US
Taliun, Daniel	University of Michigan		Ann Arbor	Michigan	48109	US
Tang, Hua	Stanford University	Genetics	Stanford	California	94305	US
Taub, Margaret	Johns Hopkins University		Baltimore	Maryland	21218	US
Taylor, Kent D.	Lundquist Institute	Institute for Translational Genomics and Populations Sciences	Torrance	California	90502	US
Taylor, Matthew	University of Colorado Anschutz Medical Campus		Aurora	Colorado	80045	US
Taylor, Simeon	University of Maryland		Baltimore	Maryland	21201	US
Telen, Marilyn	Duke University		Durham	North Carolina	27708	US
Thornton, Timothy A.	University of Washington		Seattle	Washington	98195	US
Threlkeld, Machiko	University of Washington	University of Washington, Department of Genome Sciences	Seattle	Washington	98195	US
Tinker, Lesley	Fred Hutchinson Cancer Research Center	Cancer Prevention Division of Public Health Sciences	Seattle	Washington	98109	US
Tirschwell, David	University of Washington		Seattle	Washington	98195	US
Tishkoff, Sarah	University of Pennsylvania	Genetics	Philadelphia	Pennsylvania	19104	US
Tiwari, Hemant	University of Alabama	Biostatistics	Birmingham	Alabama	35487	US
Tong, Catherine	University of Washington	Department of Biostatistics	Seattle	Washington	98195	US

Name	Institution(s)	Primary Department	Institution City	Institution State	Zip Code	Country
Tracy, Russell	University of Vermont	Pathology & Laboratory Medicine	Burlington	Vermont	05405	US
Tsai, Michael	University of Minnesota		Minneapolis	Minnesota	55455	US
Vaidya, Dhananjay	Johns Hopkins University		Baltimore	Maryland	21218	US
Van Den Berg, David	University of Southern California	USC Methylation Characterization Center	University of Southern California	California	90033	US
VandeHaar, Peter	University of Michigan		Ann Arbor	Michigan	48109	US
Vrieze, Scott	University of Minnesota		Minneapolis	Minnesota	55455	US
Walker, Tarik	University of Colorado at Denver		Denver	Colorado	80204	US
Wallace, Robert	University of Iowa		Iowa City	Iowa	52242	US
Walts, Avram	University of Colorado at Denver		Denver	Colorado	80204	US
Wang, Fei Fei	University of Washington		Seattle	Washington	98195	US
Wang, Heming	Brigham & Women's Hospital, Mass General Brigham		Boston	Massachusetts	02115	US
Wang, Jiongming	University of Michigan					US
Watson, Karol	University of California, Los Angeles		Los Angeles	California	90095	US
Watt, Jennifer	Baylor College of Medicine Human Genome Sequencing Center		Houston	Texas	77030	US

Name	Institution(s)	Primary Department	Institution City	Institution State	Zip Code	Country
Weeks, Daniel E.	University of Pittsburgh	Department of Human Genetics	Pittsburgh	Pennsylvania	15260	US
Weinstock, Joshua	University of Michigan	Biostatistics	Ann Arbor	Michigan	48109	US
Weir, Bruce	University of Washington		Seattle	Washington	98195	US
Weiss, Scott T	Brigham & Women's Hospital	Channing Division of Network Medicine, Department of Medicine	Boston	Massachusetts	02115	US
Weng, Lu-Chen	Massachusetts General Hospital		Boston	Massachusetts	02114	US
Wessel, Jennifer	Indiana University	Epidemiology	Indianapolis	Indiana	46202	US
Willer, Cristen	University of Michigan	Internal Medicine	Ann Arbor	Michigan	48109	US
Williams, Kayleen	University of Washington	Biostatistics	Seattle	Washington	98195	US
Williams, L. Keoki	Henry Ford Health System		Detroit	Michigan	48202	US
Williams, Scott	Case Western Reserve University					
Wilson, Carla	Brigham & Women's Hospital		Boston	Massachusetts	02115	US
Wilson, James	Beth Israel Deaconess Medical Center	Cardiology	Cambridge	Massachusetts	02139	US
Winterkorn, Lara	New York Genome Center		New York City	New York	10013	US
Wong, Quenna	University of Washington		Seattle	Washington	98195	US
Wu, Joseph	Stanford University	Stanford Cardiovascular Institute	Stanford	California	94305	US

Name	Institution(s)	Primary Department	Institution City	Institution State	Zip Code	Country
Xu, Huichun	University of Maryland		Baltimore	Maryland	21201	US
Yanek, Lisa	Johns Hopkins University		Baltimore	Maryland	21218	US
Yang, Ivana	University of Colorado at Denver		Denver	Colorado	80204	US
Yu, Ketian	University of Michigan		Ann Arbor	Michigan	48109	US
Zekavat, Seyedeh Maryam	Broad Institute		Cambridge	Massachusetts	02142	US
Zhang, Yingze	University of Pittsburgh	Medicine	Pittsburgh	Pennsylvania	15260	US
Zhao, Snow Xueyan	National Jewish Health		Denver	Colorado	80206	US
Zhao, Wei	University of Michigan	Department of Epidemiology	Ann Arbor	Michigan	48109	US
Zhu, Xiaofeng	Case Western Reserve University	Department of Population and Quantitative Health Sciences	Cleveland	Ohio	44106	US
Ziv, Elad	University of California, San Francisco	Medicine	San Francisco	California	94143	US
Zody, Michael	New York Genome Center		New York	New York	10013	US
Zoellner, Sebastian	University of Michigan		Ann Arbor	Michigan	48109	US
