# Supplementary material for: Clonal hematopoiesis is associated with protection from Alzheimer’s disease
Source: Nat Med. 2023 Jun 15;29(7):1662–70. doi: 10.1038/s41591-023-02397-2 (PMC10353941; doi:10.1038/s41591-023-02397-2)
Supplement: Supplementary file 2 — Reporting Summary [file 41591_2023_2397_MOESM2_ESM.pdf]

Corresponding author(s): Siddhartha Jaiswal
Last updated by author(s): Mar 28, 2023

Reporting Summary

Nature Portfolio wishes to improve the reproducibility of the work that we publish. This form provides structure for consistency and transparency in reporting. For further information on Nature Portfolio policies, see our Editorial Policies and the Editorial Policy Checklist.

Statistics

For all statistical analyses, confirm that the following items are present in the figure legend, table legend, main text, or Methods section.

n/a	Confirmed	
☐	☒	The exact sample size (*n*) for each experimental group/condition, given as a discrete number and unit of measurement
☐	☒	A statement on whether measurements were taken from distinct samples or whether the same sample was measured repeatedly
☐	☒	The statistical test(s) used AND whether they are one- or two-sided<br>*Only common tests should be described solely by name; describe more complex techniques in the Methods section.*
☐	☒	A description of all covariates tested
☐	☒	A description of any assumptions or corrections, such as tests of normality and adjustment for multiple comparisons
☐	☒	A full description of the statistical parameters including central tendency (e.g. means) or other basic estimates (e.g. regression coefficient) AND variation (e.g. standard deviation) or associated estimates of uncertainty (e.g. confidence intervals)
☐	☒	For null hypothesis testing, the test statistic (e.g. *F*, *t*, *r*) with confidence intervals, effect sizes, degrees of freedom and *P* value noted<br>*Give P values as exact values whenever suitable.*
☒	☐	For Bayesian analysis, information on the choice of priors and Markov chain Monte Carlo settings
☒	☐	For hierarchical and complex designs, identification of the appropriate level for tests and full reporting of outcomes
☐	☒	Estimates of effect sizes (e.g. Cohen's *d*, Pearson's *r*), indicating how they were calculated

Our web collection on statistics for biologists contains articles on many of the points above.

Software and code

Policy information about availability of computer code

Data collection	Flow cytometry data was collected using FACSDIVA version 8.0.1 or earlier (BD Pharmingen). Amplicon Sequencing data were collected on the MiSeq system (Illumina). scATAC-sequencing were collected on the HiSeq2500 System (Illumina).
Data analysis	Flow cytometry data were analysed using FlowJo vlO from BD Biosciences.<br><br>Amplicon Sequencing data : Sequencing reads were aligned with BWA (http://biobwa.sourceforge.net), and variant calling and annotation done with Varscan (http://varscan.sourceforge.net) and Annovar (https://annovar.openbioinformatics.org/en/latest/).<br><br>scATAC-seq data initial processing was performed with the lOX Genomics pipeline cellranger-atac version 1.1.0 and clustering was performed with the ArchR package version 1.0.1.<br><br>Code for scATAC-seq data is available here:<br>https://github.com/juliabelk/CHIP_and_AD (https://doi.org/10.5281/zenodo.7809346)<br><br>Human cohort studies analysis R packages:<br>crrSC (https://cran.r-project.org/web/packages/crrSC/index.html)<br>meta (https://cran.r-project.org/web/packages/meta/index.html)<br>cmprsk (https://cran.r-project.org/web/packages/cmprsk/index.html)<br>PowerMediation package in R (https://cran.r-project.org/web/packages/powerMediation/index.html)<br>MendelianRandomization package (https://cran.r-project.org/web/packages/MendelianRandomization/index.html)<br>MR-PRESSO (https://github.com/rondolab/MR-PRESSO)

MASS (https://cran.r-project.org/web/packages/MASS/index.html)
MR-RAPS (https://github.com/qingyuanzhao/mr.raps)

For manuscripts utilizing custom algorithms or software that are central to the research but not yet described in published literature, software must be made available to editors and reviewers. We strongly encourage code deposition in a community repository (e.g. GitHub). See the Nature Portfolio guidelines for submitting code & software for further information.

Data

Policy information about availability of data

All manuscripts must include a data availability statement. This statement should provide the following information, where applicable:

- Accession codes, unique identifiers, or web links for publicly available datasets
- A description of any restrictions on data availability
- For clinical datasets or third party data, please ensure that the statement adheres to our policy

Individual whole-genome sequencing data and individual-level harmonized phenotypes are available through restricted access via the dbGaP TOPMed Exchange Area available to TOPMed investigators. All whole exome sequencing data and phenotype data from ADSP are available on dbGaP for investigators with approved protocols through https://www.niagads.org.

Data for FHS: https://www.ncbi.nlm.nih.gov/projects/gap/cgi-bin/study.cgi?study_id=phs000974.v1.p1
Data for CHS: https://www.ncbi.nlm.nih.gov/projects/gap/cgi-bin/study.cgi?study_id=phs001368.v2.p2
Data for ADSP:https://www.ncbi.nlm.nih.gov/projects/gap/cgi-bin/study.cgi?study_id=phs000572.v8.p4

Reference genome:
hg38: https://genome.ucsc.edu/cgi-bin/hgGateway?clade=mammal&org=Human&db=hg38
hg19: https://genome.ucsc.edu/cgi-bin/hgGateway?clade=mammal&org=Human&db=hg38

Single-cell ATAC-seq data from human brain samples is available in Gene Expression Omnibus under accession GSE192838.

Human research participants

Policy information about studies involving human research participants and Sex and Gender in Research.

Reporting on sex and gender

We adjusted for self-reported sex in the association study of CHIP and AD. To discern whether there were differences based on self-reported sex, we separately performed the analyses for males and females, keeping other aspects of the analysis the same. We did not find significant differences due to the effect of CHIP in males and females.

Population characteristics

The The Cardiovascular Heart Study (CHS) is a prospective, multi-ethnic, longitudinal study of risk factors for coronary heart disease and stroke in people aged 65 and older. The subset selected for TOPMed WGS was heavily oversampled for CHD and stroke. Since vascular disease is a risk factor for dementia, we wished to exclude this as a potential confounder. Therefore, we restricted our study to those without coronary heart disease, stroke, or prior dementia. The final study sample for CHS was 743 people, 491 of these were female. The median age was 72 years at time of blood draw for WGS. There were 123 people with e2e2 or e2e3 genotype, 26 people with e2e4 genotype, 424 people with e3e4 genotype, 162 people with e3e4 genotype, and 8 people with e4e4 genotype at APOE.

The Framingham Heart Study: FHS is a single-site, prospective and population-based study that has followed participants from the town of Framingham, MA to investigate risk factors for cardiovascular diseases. The population of Framingham was almost entirely white at the beginning of the study. All participants provided written informed consent at each examination. The subset used for TOPMed WGS included modest enrichment for related persons, but was otherwise a random selection of the overall cohort. After excluding those with prior coronary heart disease, ischemic stroke, or dementia, the final study sample for FHS was 2,437 people, 1385 of these were female. The median age was 61 years at time of blood draw for WGS. There were 310 people with e2e2 or e2e3 genotype, 46 people with e2e4 genotype, 1,584 people with e3e3 genotype, 458 people with e3e4 genotype, and 39 people with e4e4 genotype at APOE.

The Alzheimer's Disease Sequencing Project (ADSP) is a collaborative effort of the National Institutes of Aging, the National Human Genome Research Institute, and the Alzheimer's community . The whole exome sequencing (WES) set of ADSP was a case-control design where cases met NINCDS-ADRDA criteria for possible, probable, or definite AD, had documented age at onset or age at death, and APOE genotyping. A case-control selection strategy was chosen that targeted cases with minimal risk as predicted by known risk factors (age, sex, and APOE) and targeted controls with the least probability of conversion to AD by age 85 years. There was no selection based on vascular or other diseases, and these phenotypes were not available for most of the cohort. After excluding those without blood DNA or known age at blood draw and further limiting to APOE e3e3 carriers, we had 1,104 AD cases and 1,446 controls who were well matched by age. The median age was 81 years at time of blood draw for WES and there were 1,458 females.

Adult Changes in Thought (ACT) is a longitudinal, community-based observational study of brain aging in participants older than 65 randomly sampled from the Group Health Cooperative (now Kaiser Permanente Washington), a health management organization in King County, Washington. Participants from ACT were sequenced as part of ADSP. A subset of participants in the study donate their brains for research upon death, and a comprehensive neuropathological exam is performed to assess for AD and related neurodegenerative disease pathologies. For decedents with post-mortem interval of less than 8 hours, a rapid autopsy is performed in which numerous samples from multiple brain regions are taken from one hemisphere and flash frozen in liquid nitrogen. For the analysis of brain samples from ACT donors, we obtained occipital cortex samples from 12

ACT brain donors (8 CHIP carriers and 4 non-carriers). Three of these also had a frozen sample from cerebellum available, and 2 had a frozen sample from the putamen. The eight CHIP carriers represented all donors known to have CHIP and with autopsy specimens available. The four without CHIP represented a random selection of the study cohort. Of the 12 donors, 2 had AD dementia, 7 were female, 10 were APOE e3e3, 2 were APOE e3e4, the median age at autopsy was 90.5 years.

Recruitment

The Cardiovascular Heart Study : A total of 2,840 samples were sequenced as part of the Trans-omics for Precision Medicine (TOPMed) project.The samples selected for whole genome sequencing as part of TOP Med were heavily oversampled for cardiovascular disease cases/

Framingham Heart Study: A total of 4,195 samples were sequenced as part of the Trans-omics for Precision Medicine (TOPMed) project Freeze 6 release. The selection of participants for sequencing was mostly a random selection of those with available DNA, but also included some related individuals for family studies.

The Alzheimer's Disease Sequencing Project (ADSP) : A total of 5,096 cases and 4,965 controls from 24 cohorts were chosen for WES. As a result of this selection strategy, cases and controls were not well-matched for age, except for carriers of APOE e3e3 genotype. In most cases, the AD diagnosis was made prior to the blood draw, however the diagnosis was usually within 5 years of the time of blood sampling for both prevalent and incident cases.

Ethics oversight

FHS and CHS : Each study received institutional certification before deposition in dbGaP, which certified that all relevant institutional ethics committees approved the individual studies and that the genomic and phenotypic data submission was compliant with all relevant ethical regulations. Secondary analysis of the dbGaP data in this manuscript was approved by the Stanford University Institutional Review Board, and this work is compliant with all relevant ethical regulations.

ADSP: Secondary analysis of the dbGaP data in this manuscript was approved by the Partners Healthcare and Stanford University Institutional Review Boards, and this work is compliant with all relevant ethical regulations.

Adult Changes in Thought (ACT) cohort: consent for brain donation was collected from each donor and the study was approved by the University of Washington Institutional Review Board and by the Kaiser Permanente Washington Institutional Review Board.

Note that full information on the approval of the study protocol must also be provided in the manuscript.

Field-specific reporting

Please select the one below that is the best fit for your research. If you are not sure, read the appropriate sections before making your selection.

☒ Life sciences ☐ Behavioural & social sciences ☐ Ecological, evolutionary & environmental sciences

For a reference copy of the document with all sections, see nature.com/documents/nr-reporting-summary-flat.pdf

Life sciences study design

All studies must disclose on these points even when the disclosure is negative.

Sample size

We examined the association between CHIP and AD in FHS and CHS using all available participants who met the inclusion criteria described above. In order to perform a power calculation for the replication study in ADSP, we had to ensure the variant allele fraction was comparable between ADSP and TOPMed for two reasons. First, the sensitivity to detect CHIP is linked to the sequencing depth, therefore the prevalence of CHIP was higher in ADSP. Second, the associations for previously studied health outcomes related to CHIP are dependent on clone size, with small clones having less of an effect size. We empirically determined that a cutoff of VAF at 0.08 gave a nearly identical VAF distribution for CHIP clones in ADSP as compared to TOPMed. After these exclusions, we had 2,550 persons in ADSP for the analysis, of whom 43% were AD cases and 17% were CHIP carriers at a VAF>0.08. We then used the powerMediation (https://cran.r-project.org/web/packages/powerMediation/index.html) package in R to perform a power calculation for varying effect sizes of CHIP at an alpha of 0.1. For an odds ratio of 0.6 (similar to the hazard ratio for CHIP obtained from TOPMed), the power was 1. For an odds ratio of 0.8, the power was 0.96. For an odds ratio of 0.9, the power was 0.50. Thus, we were well-powered for the replication analysis in ADSP.

For the analysis of CHIP and neuropathology, we used all available data from people without dementia in ADSP. For the Mendelian randomization analysis, we used all publicly available AD GWAS or GWAX summary statistics.

For the analysis of CHIP variants in brain, we used all available donor samples that had CHIP in ACT. For the selection of samples for scATACseq, we included the 6 samples where CHIP variants could be detected in unsorted brain. An additional 4 brain samples from non-CHIP carriers were included as controls. Power calculations were not performed to pre-determine sample size in these analyses.

Data exclusions

In FHS and CHS, we excluded anyone with a diagnosis of coronary heart disease and stroke to avoid confounding from vascular dementia. In ADSP, carriers of APOE e2 or APOE e4 alleles were selected in such a way that cases and controls were poorly matched for age. Due to this selection bias, carriers of these alleles were excluded from the analysis. However, APOE e3e3 carriers were well matched for age , allowing for us to use this set as the replication cohort. In all studies, we excluded anyone with missing information on age at blood draw.

Replication

All attempts at replication were successful.

	The association between CHIP and AD dementia in TOPMed was replicated in ADSP, and similar observations were made in the analysis of CHIP and neuropathology and in Mendelian randomization analysis. Thus, all attempts at replication of this association were successful. The association of CHIP and AD dementia stratified by CHIP status in TOPMed was replicated in ADSP samples without dementia using neuropathology.<br><br>For the amplicon sequencing of CHIP variants from brain, we replicated the sorting and sequencing in 4 samples, and we replicated the sequencing results in unsorted brain in 7 samples.<br><br>For the single-cell ATAC analysis of brain, we first performed this on samples from 2 CHIP carriers. A second set of 4 CHIP carriers replicated the main findings that mutant cells resembling microglia could be found in brains of CHIP carriers.
Randomization	Randomization was not applicable here as we used previously collected data and biosamples for association studies and for analysis of human brain tissue.
Blinding	The sequencing data was generated and phenotype collection was done by researchers with no knowledge of whether the participants had CHIP. The assessment of CHIP from TOPMed and ADSP sequencing data was done prior to any knowledge of the AD phenotype data. Blinding was not possible for association analyses of CHIP and AD because investigators analyzing the data would need to know the status of all the variables used in the analysis. Investigators analyzing the scATACseq data were blinded to the CHIP status of the samples at time of analysis.

Reporting for specific materials, systems and methods

We require information from authors about some types of materials, experimental systems and methods used in many studies. Here, indicate whether each material, system or method listed is relevant to your study. If you are not sure if a list item applies to your research, read the appropriate section before selecting a response.

Materials & experimental systems

n/a	Involved in the study
☐	☒ Antibodies
☒	☐ Eukaryotic cell lines
☒	☐ Palaeontology and archaeology
☒	☐ Animals and other organisms
☐	☒ Clinical data
☒	☐ Dual use research of concern

Methods

n/a	Involved in the study
☒	☐ ChIP-seq
☐	☒ Flow cytometry
☒	☐ MRI-based neuroimaging

Antibodies

Antibodies used	Anti-NeuN Antibody Alexa Fluor 488, clone A60. EMD Millipore MAB377X<br>Anti-MAF antibody PE, clone T54853 .BD biosciences 565795
Validation	Anti-NeuN Antibody Alexa Fluor 488 (Clone A60,MAB377X). Validation by the manufacturer that states: Clone A60 detects level of NeuN and has been published and validated for use in FC, IC, IF, IH, IH(P), IP and WB. We tested and validated it for flow cytometry by testing different concentrations on the experimental samples. The concentration used for the experiments is 1:400.<br>Specificity of the antibody was shown in this publication "Rbfox Splicing Factors Promote Neuronal Maturation and Axon Initial Segment Assembly", PMID: 29398366. Where the antibody was used to detect Rbfox3 protein in motor neurons depleted or not for Rbfox3 protein and allowed the knock out validation.<br><br>Anti-MAF antibody PE for flow cytometry was validated for intracellular staining of the Transcription Factor C-MAF by BD Biosciences on Human peripheral blood mononuclear cells (PBMC). We tested and validated it for flow cytometry by testing different concentrations on the experimental samples. the concentration used for the experiments is 1:50.

Clinical data

Policy information about clinical studies

All manuscripts should comply with the ICMJE guidelines for publication of clinical research and a completed CONSORT checklist must be included with all submissions.

Clinical trial registration	*Provide the trial registration number from ClinicalTrials.gov or an equivalent agency.*
Study protocol	*Note where the full trial protocol can be accessed OR if not available, explain why.*
Data collection	*Describe the settings and locales of data collection, noting the time periods of recruitment and data collection.*
Outcomes	*Describe how you pre-defined primary and secondary outcome measures and how you assessed these measures.*

Flow Cytometry

Plots

Confirm that:

☒ The axis labels state the marker and fluorochrome used (e.g. CD4-FITC).

☒ The axis scales are clearly visible. Include numbers along axes only for bottom left plot of group (a 'group' is an analysis of identical markers).

☒ All plots are contour plots with outliers or pseudocolor plots.

☒ A numerical value for number of cells or percentage (with statistics) is provided.

Methodology

Sample preparation	Around 250 mg of frozen postmortem brain tissue was thawed in 5 ml lysis buffer and transferred to a douncer placed on ice. After 20-30 strokes, the homogenized tissue was transferred to a clear 50 ml ultracentrifuge tube and the volume was adjusted to 12 ml. 21 ml of sucrose buffer was added to the bottom of the clear ultracentrifuge tube, to create a concentration gradient with the homogenized tissue solution on top of the sucrose buffer. The tubes were placed in buckets in a SW32Ti swinging rotor (Beckton Dickinson). The samples were ultracentrifuged at 107163G for 2.5 hours at 4 C. The supernatant was removed and 500 µl of lX PBS was added to the pellet and incubated for 20 min on ice. The nuclei were then resuspended and transferred into a microcentrifuge tube. The nuclei were counted using trypan blue dilution and then centrifuged at S00G for 5 min.<br>The nuclei were resuspended at a concentration of 200,000 cells in 50ul of 0.5% BSA in lx PBS solution and stained for 45 min with Anti-NeuN Antibody Alexa Fluor 488(EMD Millipore) at a concentration of 1: 400, and Anti-C-MAF antibody PE (BD biosciences) at a concentration of 1: 50. The nuclei were then washed and strained using a 40um strainer. The sorting was done on an Aria II sorter using a lO0um nozzle. The nuclei were collected in 0.5% BSA in lx PBS solution and centrifuged at S00G for 5 min.
Instrument	The sorting was done on an BD Aria II sorter
Software	FlowJo vlO from BD Biosciences
Cell population abundance	Populations of interest were sorted to >98% purity, as measured by flow cytometric analysis of sorted populations.
Gating strategy	For all experiments, the single cell population which formed the basis for further gating was defined based on subsequent FSC-A/SSC-A, FSC-A/FSC-H, and SSC-W/SSC-H gates. For all markers (NeuN and C-Maf) for sorting, positive and negative gates were defined by the bimodal distribution of the fluorescence signal.

☒ Tick this box to confirm that a figure exemplifying the gating strategy is provided in the Supplementary Information.
